# Type II Restriction of Bacteriophage DNA With 5hmdU-Derived Base Modifications

**DOI:** 10.3389/fmicb.2019.00584

**Published:** 2019-03-29

**Authors:** Kiersten Flodman, Rebecca Tsai, Michael Y. Xu, Ivan R. Corrêa, Alyssa Copelas, Yan-Jiun Lee, Ming-Qun Xu, Peter Weigele, Shuang-yong Xu

**Affiliations:** New England Biolabs, Inc., Ipswich, MA, United States

**Keywords:** Type II restriction and modification, 5hmdU-derived nucleotide modification, *Pseudomonas* bacteriophage (phage) M6, *Salmonella* phage ViI, *Delftia* phi W-14, phage therapy

## Abstract

To counteract bacterial defense systems, bacteriophages (phages) make extensive base modifications (substitutions) to block endonuclease restriction. Here we evaluated Type II restriction of three thymidine (T or 5-methyldeoxyuridine, 5mdU) modified phage genomes: *Pseudomonas* phage M6 with 5-(2-aminoethyl)deoxyuridine (5-*N*edU), *Salmonella* phage ViI (Vi1) with 5-(2-aminoethoxy)methyldeoxyuridine (5-*N*e*O*mdU) and *Delftia* phage phi W-14 (a.k.a. ΦW-14) with α-putrescinylthymidine (putT). Among >200 commercially available restriction endonucleases (REases) tested, phage M6, ViI, and phi W-14 genomic DNAs (gDNA) show resistance against 48.4, 71.0, and 68.8% of Type II restrictions, respectively. Inspection of the resistant sites indicates the presence of conserved dinucleotide TG or TC (TS, S=C, or G), implicating the specificity of TS sequence as the target that is converted to modified base in the genomes. We also tested a number of DNA methyltransferases (MTases) on these phage DNAs and found some MTases can fully or partially modify the DNA to confer more resistance to cleavage by REases. Phage M6 restriction fragments can be efficiently ligated by T4 DNA ligase. Phi W-14 restriction fragments show apparent reduced rate in *E. coli* exonuclease III degradation. This work extends previous studies that hypermodified T derived from 5hmdU provides additional resistance to host-encoded restrictions, in parallel to modified cytosines, guanine, and adenine in phage genomes. The results reported here provide a general guidance to use REases to map and clone phage DNA with hypermodified thymidine.

## Introduction

Type II restriction and modification (R-M) systems in bacteria encode restriction endonucleases (REases) to destroy invading foreign DNA in phage infection and acquisition of mobile genetic elements (Smith and Wilcox, 1970; Landy et al., 1974, reviewed in Pingoud et al., 2016). To gain an upper hand in the biowarfare, bacteriophages (phages) utilize DNA base modifications [or nucleotide (nt) substitutions] to counteract host-encoded Type II restrictions (Huang et al., 1982; Kruger and Bickle, 1983; Miller et al., 1985; Kulikov et al., 2014; Tsai et al., 2017; Lee et al., 2018). Extensive non-canonical nt substitutions have been reported for all four bases in DNA: for example, 5-methylcytosine (5mC) replacing all C in phage XP12 genome (Feng et al., 1978), 5-glucosylated-hydroxymethylcytosines (5gmC) in phage T4 (Gold and Schweiger, 1969), deoxyarchaeosine (dG^+^) in phage 9g (Thiaville et al., 2016; Tsai et al., 2017), α-putrescinylthymidine (putT) in phi W-14 (Kelln and Warren, 1973; Kropinski et al., 1973), *N*6-methyladenine (6mA) in some phages encoding frequent adenine methyltranferases (MTases) (Drozdz et al., 2012; Murray et al., 2018), and 5-hydroxymethyluridine (5hmdU) in *Bacillus* phage SP8 and SPO1 (Stewart et al., 2009, reviewed in Weigele and Raleigh, 2016). Non-canonical nt substitutions can be introduced during DNA replication through modified dNTP (e.g., 5hmCTP, 5hmdUTP). Further base modification can be carried out post-replicationally by phage-encoded enzymes such as MTases, DNA glycosyltranferases, and alkylamine transferases. In some cases, phage DNA is partially modified by host-encoded enzymes, such as Dcm (methylation of CCWGG to C5mCWGG) and Dam methyltransferases (methylation of GATC to G6mATC), or other MTases during phage DNA replication. Phage T4 DNA modified with 5gmC is resistant to many Type II REases that recognize GC-containing sequences (Huang et al., 1982). Phage 9g DNA with the dG^+^ modification is resistant to ∼71% of Type II restriction specificities with GC sequences (Tsai et al., 2017). In a limited Type II restriction study of phage phi W-14 genome containing putT, 17 out of 32 REases tested were blocked by the base modification (Miller et al., 1985). The additional positive charges from the side chain of putT likely interfere with restriction enzyme tracking process due to altered local DNA structure since the cleavage efficiency of some REases with only GC sequence was also impaired. Phi W-14 genomic DNA is packaged more compactly in phage head than T4 (Scraba et al., 1983). Phage SPO1 genomic DNA with 5hmdU substitutions is fully resistant to 4 out of 30 Type II restrictions (∼13.3%) and partially resistant (i.e., slower cleavage in 1 h digestion) to 17 out of 30 REases tested (56.7%) (Huang et al., 1982; Vilpo and Vilpo, 1995). DNA duplex oligos with the 5hmdU substitution display reduced melting temperature (Tm) and altered backbone flexibility when passing through nanopores (Carson et al., 2016).

Two new base modifications, 5-(2-aminoethyl)deoxyuridine (5-*N*edU) and 5-(2-aminoethoxy)methyldeoxyuridine (5-*N*e*O*mdU) were recently discovered in the genomes of *Pseudomonas* phage M6 and *Salmonella* phage ViI (Vi1) (Lee et al., 2018). Hypermodified *Pseudomonas* phage DNAs were shown to be resistant to Type II restriction. Phage M6 and ViI encode a modification gene cluster in their genomes for the production of 5hmdU and the enzymes responsible for subsequent reactions to add the desired chemical groups (Lee et al., 2018). It has been predicted that these phages also encode their own primase, DNA polymerase/clamp loader protein/sliding clamp holder protein, DNA ligase, and RNase H, all of which displaying specialized properties to incorporate modified dNTP intermediate during replication. The three phages M6, ViI, and phi W14 containing hypermodified thymidine bases are thought to utilize the common intermediate 5hmdU. 5hmdU is incorporated into DNA, then phosphorylated by a 5hmdU DNA kinase, and further modified by alkylamine transferases and other associated enzymes. Not all thymidines in the genome are replaced by 5hmdU; in addition to the hypermodified base, these phage DNAs may also carry regular base T and 5hmdU. Bioinformatic prediction of enzymes involved in phage nucleotide hypermodifications has provided abundant information on gene clusters and biosynthetic pathways (Iyer et al., 2013).

The goal of this work is to examine Type II restrictions of modified DNA in phage M6, ViI, and phi W-14 genomes. We performed restriction digestions of these three gDNAs to verify their resistant level *in vitro*. We also analyzed the resistant sites for any conserved sequence motifs to shed light on possible modification site specificity. Furthermore, we introduced additional base modifications in their DNA by treatment with cytosine or adenine MTases to generate two types of base modifications (for instance in M6 DNA, a combination of 5mC and 5-*N*edU, or 6mA and 5-*N*edU). We also examined the ligation efficiency of phage DNA restriction fragments and tested two exonuclease activity on the modified DNA. This work provides basic information on restriction of T-modified DNA and further our understanding of the co-evolution relationship of host and hypermodified phage genomes. Study of highly modified phage genomes may have impact in phage therapy.

## Materials and Methods

### Phage DNA Purification and Restriction Digestions

REases, MTases, DNA ligase, DNA nuclease, and phosphatase, Proteinase K, exonuclease, and repair enzyme hSMUG1 were provided by New England Biolabs (NEB). Phage particles were purified by CsCl gradient method and phage DNA purified by phenol-CHCl_3_ extraction, and ethanol precipitation (Sambrook et al., 1989). Due to poor phage titer of M6 phage, phage infection and propagation were carried out on solid growth medium and phage lysates were pooled from multiple plates. NEBcutter V2.1 software (Vincze et al., 2003) was used to generate restriction patterns of phage DNA with the assumption of no base modification. We used excess of REases in restriction digestions (5 to 40 U to cleave 0.25 to 0.5 μg phage DNA) in 50 μl total volume incubated at the recommended temperature for 1 h (e.g., 5 μl of REases for low concentration enzyme supplied at 1000 U/ml, 2 μl of REase for high concentration REase supplied at 20,000 U/ml). Digested DNAs were analyzed by agarose gel electrophoresis. The DNA cleavage patterns were compared to NEBcutter-generated restriction patterns to determine digestion results as complete (c), partial (p), very partial (vp), or resistant (x) to digestions. For digestion of viral DNA with glycosylase and AP endonuclease, DNA was first incubated with hSMUG1 for 1 h, and then treated with *Escherichia coli* endonuclease VIII.

### Methylation and Challenge With REases to Check Methylation Level

Phage DNA was methylated by treatment with excess DNA MTase and methyl-donor SAM in the recommended buffer for 2 h. Following Proteinase K treatment and spin column purification, the methylated DNA was digested by cognate or non-cognate REases to evaluate the degree of resistance to restriction.

### Methylation and Determination of Base Compositions by Liquid Chromatography-Mass Spectrometry (LC-MS)

Phage DNA was methylated by the frequent MTases M.EcoGII (adenine methyltransferase), M.SssI (CpG methyltransferase), M.CviPI (GpC methyltransferase) for 2–4 h with methyl donor SAM. After Proteinase K treatment, the DNA was precipitated in ethanol, dried and resuspended in a buffer for nuclease degradation. DNA samples (5 μg) were digested to nucleosides by treatment with the Nucleoside Digestion Mix (NEB, M0649S) overnight at 37°C. Nucleoside analysis was performed on an Agilent LC/MS System 1200 Series instrument equipped with a G1315D diode array detector and a 6120 Single Quadrupole Mass Detector operating in positive (+ESI) and negative (-ESI) electrospray ionization modes. LC was carried out on a Waters Atlantis T3 column (4.6 mm × 150 mm, 3 μm) with a gradient mobile phase consisting of 10 mM aqueous ammonium acetate (pH 4.5) and methanol. MS data acquisition was recorded in total ion chromatogram (TIC) mode. Each nucleoside was identified as follows: dC [M + H]^+^ 228.1 and [M-H]^-^ 226.2; dG [M + H]^+^ 268.1 and [M-H]^-^ 266.1; dT [M + H]^+^ 243.1 and [M-H]^-^ 241.1; dA [M + H]^+^ 252.1 and [M-H]^-^ 250.1; 5mdC [M + H]^+^ 242.1 and [M-H]^-^ 240.2; 6mdA [M + H]^+^ 266.1 and [M-H]^-^ 264.1; 5hmdC [M-H]^-^ 257.0; 5-*N*e*O*mdU [M + H]^+^ 302.1 and [M-H]^-^ 300.1; α-putT [M + H]^+^ 329.2 and [M-H]^-^ 327.2; putT-dC [M + H]^+^ 618.3 and [M-H]^-^ 616.2; and putT-dG [M + H]^+^ 658.2 and [M-H]^-^ 656.1. The relative abundance of each nucleoside was determined by dividing the UV absorbance by the corresponding extinction coefficient at 260 nm.

## Results

### Restriction of Phage M6, ViI, and phi W-14 Genomic DNA

**FIGURE 1:**
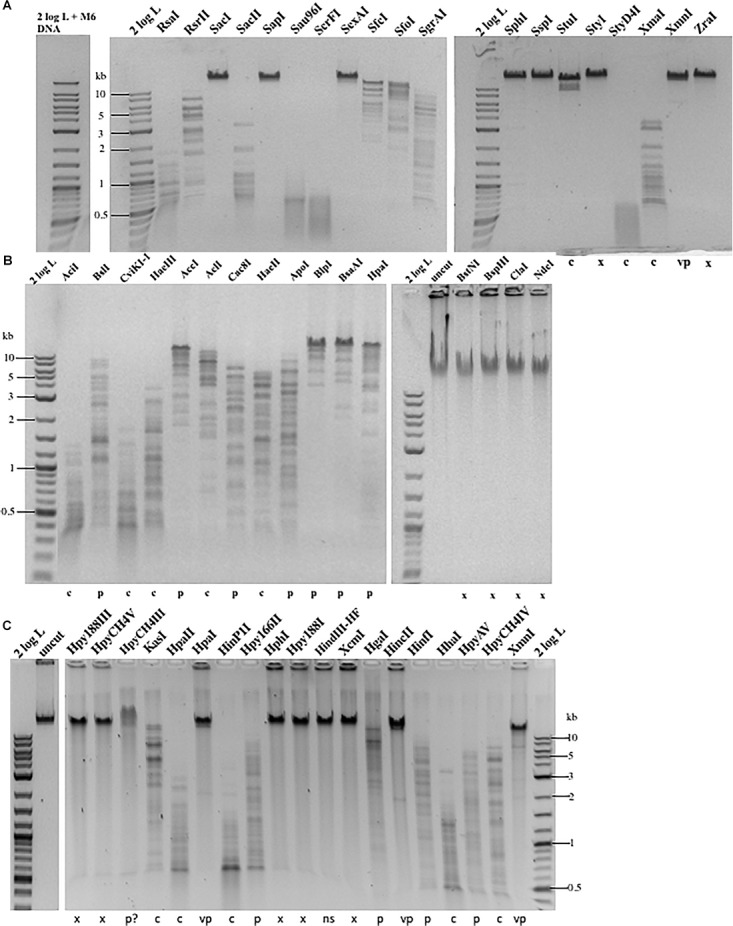
Representative examples of Type II restriction of phage gDNA. **(A)** Phage M6 DNA digested with 19 REases and analyzed by agarose gel electrophoresis. X, resistant to restriction; C, complete digestion; C^∗^, additional fragments observed owing to star activity; P, partial digestion; VP, very partial digestion (only a few weak bands detected); 2 log DNA ladder (0.1–10 kb). Phage M6 DNA is resistant to restriction by *Sac*I (GAGCTC), *Sap*I (GCTCTTC), *Sex*AI (ACCTGGT), *Sph*I (GCATGC), *Ssp*I (AATATT), *Sty*I (CCATGG), *Xmn*I (GAAN4TTC), and *Zra*I (GACGTC), likely due to the presence of TS dinucleotides in one (*Sap*I) or both strands. *Ssp*I site with six Ts in the recognition sequence is also resistant. The restriction results are summarized in Supplementary Table 1. The computer-generated restriction patterns by NEBCutter are shown in Supplementary Figures 3–5. **(B)** Representative examples of restriction digestions of phage ViI gDNA. The ViI DNA is resistant to restriction by *Bst*NI (CCTGG), *Bsp*HI (TCATGA), *Cla*I (ATCGAT), and *Nde*I (CATATG), likely due to the presence of modified T in TS (TG or TC) dinucleotides in one or both strands. The restriction results are summarized in Supplementary Table 2. **(C)** Representative examples of phi W-14 gDNA digested by 19 REases. X, resistant; C, complete digestion; P, partial digestion; VP, very partial digestion; NS, no restriction sites present (as internal negative control); P?, DNA bound and slightly shifted with some smearing; Phi W-14 DNA is resistant to restrictions by *Hpy*188III (TCNNGA), *Hpy*CH4V (TGCA), *Hpa*I (GTTAAC), *Hph*I (GGTGA), *Hpy*188I (TCNGA), *Xcm*I (CCAN9TGG), *Hin*cII (GTYRAC), *Hin*fI (GANTC), and *Xmn*I (GAAN4TTC). The phi W-14 DNA is partially resistant to HpyCH4III (ACNGT) since the probability of having TS sequence with immediate flanking 3′ nt in ACNGTS is 0.5 and the chance of having ACTGT is 0.25. The phage DNA is largely resistant to *Hpa*I (GTTAAC) with tG and 4Ts in both strands. The restriction results are summarized in Supplementary Tables 1–3.

To find out the resistance level, we carried out restriction digestions for phage M6, ViI, and phi W-14 genomic DNA. The chemical structure of the modified bases discussed in this work is shown in Supplementary Figure 1. It was unknown beforehand how many units are required for complete digestion of each modified DNA since the unit definition is typically done on phage λ or pBR322 DNA by the manufacturer. We used phage λ and pTYB2 DNA for control digestions to validate REases that are active, but not able to cleave modified DNA. The restriction of modified phage DNA was repeated at least once to confirm reproducibility. We grouped restriction results into four categories: complete, partial, very partial (most of the substrate DNA remains intact, only a few weak bands visible), and resistant as compared to computer generated banding patterns. The results are shown in Figure 1A–C and Table 1. Phage M6, ViI, and phi W-14 DNAs are resistant to approximately 48.4, 71.0, and 68.8% of Type II restrictions, as compared to phage 9g DNA (dG^+^ modification) resistance to nearly 71% REases tested. The individual restriction results for three genomic DNAs are shown in Supplementary Tables 1–3. Phage M6 DNA is completely resistant to *Fsp*I (TGCGCA) and *Sac*I (GAGCTC) restriction, most likely due the modified T in TG and TC dinucleotide in both strands (see below for more resistant site analysis). Phage ViI DNA is resistant to restriction by *Bsp*HI (TCATGA), *Cla*I (ATCGAT), and *Nde*I (CATATG). Phi W-14 DNA is resistant to restriction by *Hpy*188III (TCNNGA) and *Hpy*CH4V (TGCA) probably due to the modified bases in TG or TC dinucleotides in both strands. In some cases, phage DNA is also partially or completely resistant to REases that cleave target sites with 4–6 AT bp (see Supplementary Tables 1–3). We concluded that the longer side-chain modifications of phages ViI and phi W-14 DNAs are more effective at blocking Type II restriction than is the smaller aminoethyl group of phage M6 DNA. However, 5-*N*edU shows better resistance than phage DNA with 5hmdU alone (Vilpo and Vilpo, 1995). The partial positive charges of the side chain in the major groove of DNA may affect the indirect read out of target sequence by REases. The phage DNA sensitivity to Type II restriction is also shown in “pie” charts (Supplementary Figure 2). Since most of the restriction reactions were carried out with excess enzymes in an overdigestion protocol, we cannot rule out the possibility that some very partial digestions are caused by relaxed “star” activity (restriction enzyme “star” activity can cleave target sites with 1–2 bp off from the canonical sites) (Robinson and Sligar, 1993). Engineered high-fidelity REases were used where available to minimize “star” activity (Vasu et al., 2013). Thus, the resistance level might be underestimated compared to the *in vivo* restriction level. *In vivo* restriction gene expression is tightly regulated by transcription factors such as the C (controller) protein to prevent self-restriction (Tao and Blumenthal, 1992; Sawaya et al., 2013).

**Table 1:** Type II restriction of phage M6, ViI, and phi W-14 genomic DNA.

Cleavage status	M6	ViI	Phi W-14
Complete	33.7%	8.3%	10.7%
Inconclusive^∗^	1.6%	2.7%	0.9%
Partial	16.3%	18.0%	19.6%
Very partial^∗∗^	7.9%	6.9%	8.4%
Resistant	40.5%	64.1%	60.4%
Very partial + resistant	48.4%	71.0%	68.8%


*^∗^Inconclusive: restriction fragments too large (>10 kb) to be clearly resolved in 0.8–1% agarose gel. ^∗∗^Very partial: most of the genomic DNA remains intact and only a few weak bands were detected.*

There are a number of REases that recognize and cleave target sites with GC bp sequence only. Interestingly, they can cut λ and plasmid (pTYB2) DNA; but are unable to cleave M6 and phi W-14 DNA (Supplementary Figures 6A,B). We speculate that these REases are extremely sensitive to the nearby base modifications since the probability of TG dinucleotide 5′ to the *Apa*I (GGGCC/C) and *Psp*OMI (G/GGCCC) sites is only 0.25. Similarly, the probability of TG dinucleotide 5′ to the NarI (GG/CGCC) and PluTI (GGCGC/C) is 0.25. NarI and PluTI partially digested a single site plasmid pTYB2 as two sites are probably required for efficient digestion. This group of enzymes include Type IIE and IIF that requires a secondary site (effector site) and extensive looping and enzyme complex interaction (enzyme dimers or tetramers bound to two sites separated by a certain distance) (Roberts et al., 2003). Phi W-14 genomic DNA is resistant or partially resistant to *Apa*I, *Nae*I (GCC/GGC), *Ngo*MIV (G/CCGGC), *Not*I (GC/GGCCGC), or *Psp*OMI digestion (Supplementary Figure 6B). The presence of TG dinucleotides (e.g., tGCCGGC) in the flanking sequence may play a role in the resistance, but it cannot explain all resistant sites.

### Conserved Sequence Motif Among the Resistant Sites in Phage DNA

It has been proposed that M6, ViI, and phi W-14 phages utilize phage-encoded DNA polymerases and a 5hmdUTP, dATP, dCTP, and dGTP deoxynucleotide pool for DNA replication, thus replacing all T with 5hmdU (Neuhard et al., 1980). Further base modifications can occur post-replicationally on the hydroxymethyl moiety of 5hmdU via a phosphorylated intermediate by the action of a 5hmdU DNA kinase (5-HMUDK). It is not known whether the modification site is random or has certain sequence specificity. When the resistant sites were analyzed we observed a predominant sequence motif of TG, TC, TG+TC, or TS+TN dinucleotide. Table 2 shows that 44 out of 77 resistant sites (57.1%) contain a TG, TC, or TG+TC sequence in phage M6 DNA, while 58.3% of the resistant sites in phage ViI contain the TS motif. The frequency of TS sequence in resistant sites is slightly lower in phage phi W-14 DNA at 49.6%. These numbers are probably underestimated since they do not include the flanking sequence T outside of restriction sites (for example tGCCGGC with a TG dinucleotide). In the restriction analysis of these three phage DNAs, the majority of the resistant sites contain TS dinucleotide in combination with another TT or TA dinucleotide. This suggests that the 5hmdU DNA kinase involved in the phosphorylation of 5hmdU very likely shows the same preference for the TG or TC (TS) sequences. Consistent with the above observation, purified 5hmdU DNA kinase from phage M6 can modify TG dinucleotides in phage SP8 genomic DNA containing 5hmdU (PW, unpublished result).

**Table 2:** Frequency of dinucleotide in the resistant sites among the phage genomic DNA.

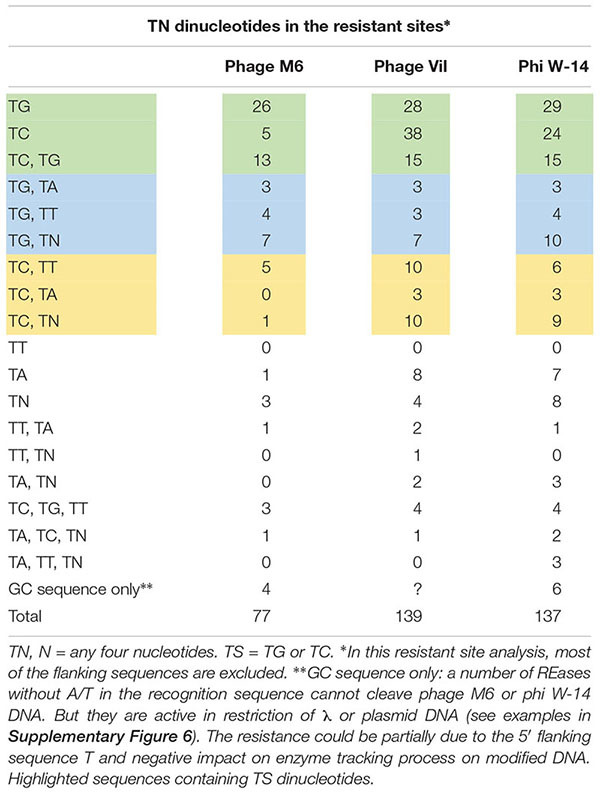

### Methylation of Phage M6 DNA to Generate 5mC- or 6mA-Modified DNA

**FIGURE 2:**
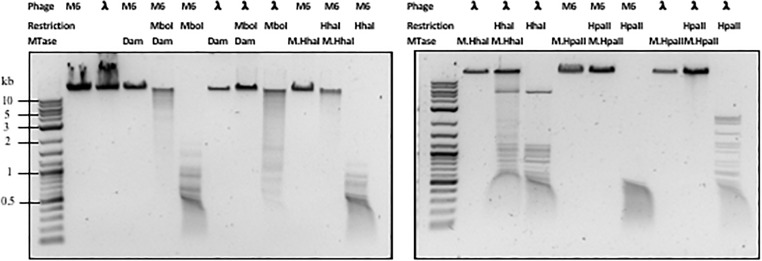
Methylation and restriction of phage M6 gDNA. Phage M6 and λ DNAs were methylated by incubation with indicated MTase and then challenged with cognate or non-cognate REase. Partial restriction digestions resulted from partial modification by the MTase. Resistance to digestions indicate full modification by the MTase.

Next, we examined whether *Pseudomonas* phage M6 DNA can be further modified by C5-cytosine and adenine MTases. This experiment has two possible outcomes: (1) if M6 DNA can be further modified by these MTases, the double-modified DNA may be resistant to more Type II restrictions; (2) 5mC- and N6mA-modified DNA may be subjected to 5mC-dependent restriction systems such as *Eco*K *Mcr*BC (Dila et al., 1990) and *Mcr*A (Mulligan and Dunn, 2008; Czapinska et al., 2018) or N6mA-dependent restriction system *Eco*K Mrr (Heitman and Model, 1987) and Pgl system found in *Streptomyces*, respectively (Hoskisson et al., 2015). Host-acquired modifications of phage genome by Type I MTases were discovered in the early days of molecular biology and phage genetics (Luria and Human, 1952; Bertani and Weigle, 1953; Luria, 1953). Over-expression of a Type II DNA MTase M.BsuM partially modified phage SP10 genome and increased the phage plating efficiency on restriction-proficient (BsuMR^+^) strain (Matsuoka et al., 2005). In this work we performed DNA methylation and subsequent restriction *in vitro*. After methylation reactions and proteinase K treatment, the phage M6 DNA was purified by spin column and subjected to restriction by the cognate REase or non-cognate endonuclease that is supposed to be blocked by the methylation. Figure 2 shows representative examples of methylation and restriction experiments. After M.CviPI (GpC methyltransferase) or M.HhaI methylation, the M6 DNA is largely resistant to *Hha*I (GCGC) restriction. M.HpaII and M.MspI can also fully modify M6 DNA and render the DNA resistant to *Hpa*II (CCGG) and *Msp*I (CCGG) restriction, respectively. M.SssI and M.AluI can partially modify the M6 DNA and provide partial resistance to *Hpa*II or *Alu*I (ACGT) restrictions. For the adenine MTases, Dam methyltransferase, M.EcoGII, and M.TaqI can partially modify the M6 DNA and provide partial resistance to *Mbo*I (GATC) and *Taq*I (TCGA) restriction. The methylation and restriction results are summarized in Table 3. We concluded that phage M6 DNA can be further modified by C5-cytosine or adenine MTases, which provide additional protection against Type II restriction. The secondary nt modifications might be beneficial for using *Pseudomonas* lytic phages to combat multi-drug resistant *Pseudomonas* infection.

**Table 3:** Methylation and subsequent restriction challenge of methylated phage M6 DNA.

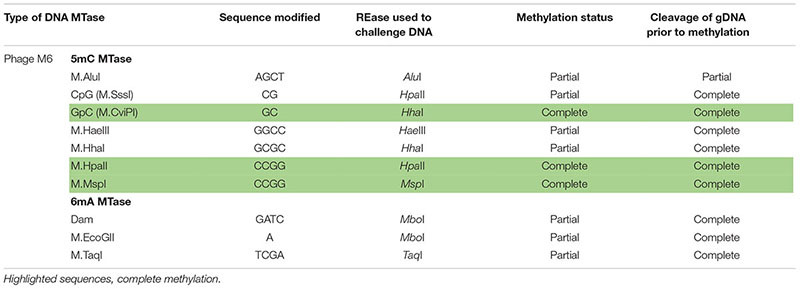

### Base Composition Analysis of Methylated Phages ViI and phi W-14 DNA

**FIGURE 3:**
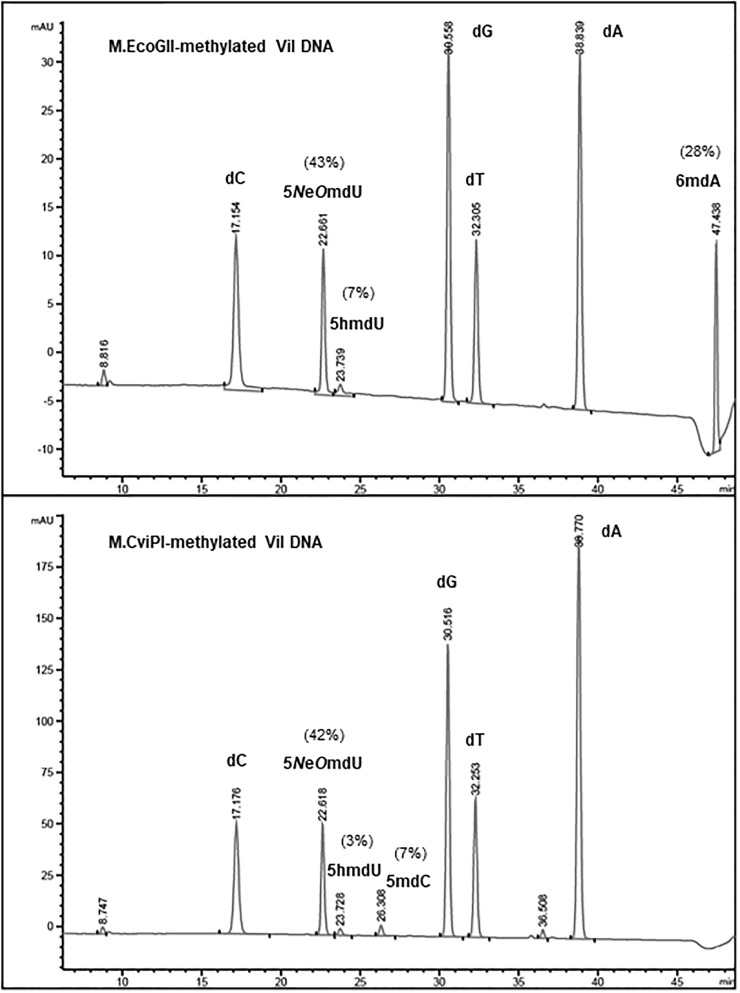
Base composition analysis by LC-MS of M.EcoGII- or M.CviPI-methylated ViI gDNA. The distribution of thymine-derived bases 5-*N*e*O*mdU, 5hmdU, and dT were estimated at 43, 7, and 50% in phage ViI genome, respectively. The percentage of 6mA generated by treatment with M.EcoGII was approximately 28% of the total adenosines (**top** panel). The percentage of 5mC was only 7% after methylation of the phage ViI gDNA with the GpC methyltransferase M.CviPI (**bottom** panel).

To estimate the level of methylation in phage ViI and phi W-14 DNAs, we performed LC-MS analysis of the corresponding MTase-treated DNAs. Figure 3 shows that ∼28% of adenosines have been methylated to 6mA in M.EcoGII-treated ViI genomic DNA. M.CviPI-treated ViI DNA gave rise to ∼7% of 5mC. The composition of the naturally occurring 5-*N*e*O*mdU, 5-hmdU, and T in phage ViI genomic DNA were estimated at 43, 7, and 50%, respectively. In a control experiment, M.EcoGII-mediated A to 6mA conversion and M.CviPI-mediated GpC to Gp5mC conversion in phage λ DNA reached ∼93 and ∼30%, respectively (data not shown).

**FIGURE 4:**
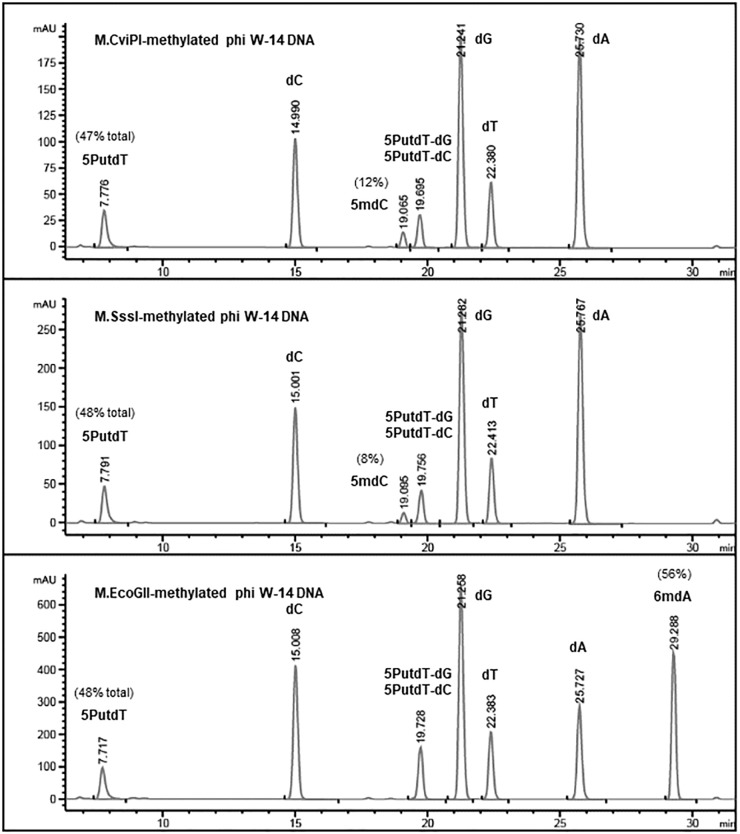
Base composition analysis by LC-MS of M.CviPI-, M.SssI-, and M.EcoGII-methylated phi W-14 genomic DNA. The percentage of 5mC was ∼8 and ∼12% after treatment with the GpC and CpG methyltransferases M.CviPI and M.SssI, respectively. 6mA levels reached 56% after methylation with M.EcoGII. The natural modified base putT was detected in the range of 47–48%, in close agreement with previously published results (∼50%). A small fraction of putT was present in the form putT-G and putT-C dinucleotides due to incomplete digestion of the phi W-14 DNA.

Base composition analysis of the *Eco*GII-treated phi W-14 genomic DNA indicated that 56% of adenosines were converted to 6mA (Figure 4). The C5-cytosine MTases M.CviPI and M.SssI were capable of converting 8% and 12% of cytidines to 5mC in phi W-14 DNA, respectively. The naturally occurring putT in phage phi W-14 DNA was detected at approximately 48%, which is consistent with the ∼50% putT reported in a previous work (Kropinski et al., 1973; Maltman et al., 1980) (note that total levels of putT reported here include the putT-G and putT-C dinucleotides, which result from the incomplete digestion due to the presence of the putrescinyl group) (Figure 4). The reason for poor methylation by the C5 MTases on ViI and phi W-14 DNA is unknown. Poor cytosine methylation may provide certain advantage against 5mC-dependent restriction systems such as *Bis*I, *Mcr*BC, *Mcr*A, *Msp*JI, and *Taq*I homologs (Cohen-Karni et al., 2011; Xu et al., 2016; Kisiala et al., 2018).

### Ligation of Restriction Fragments of Phages ViI and phi W-14 DNA

**FIGURE 5:**
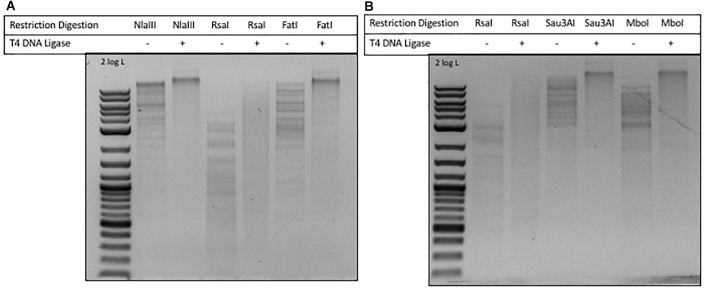
Ligation of phages ViI and phi W-14 restriction fragments by T4 DNA ligase. **(A)** Ligation of phage ViI restriction fragments partially digested by *Nla*III and *Fat*I, and completely digested by *Rsa*I. Cleavage sites of REases: *Nla*III (CATG/), 3′ 4-base overhang; *Rsa*I (GT/AC), blunt ends; *Fat*I (/CATG), 5′ 4-base overhang. **(B)** Ligation of phage phi W-14 restriction fragments generated by *Rsa*I, *Sau*3AI, and *Mbo*I digestions. Cleavage sites of *Rsa*I (GT/AC), blunt ends; *Sau*3AI (/GATC), and *Mbo*I (/GATC), 5′ 4-base overhang.

In phage ViI DNA, approximately 43% of Ts have been replaced by 5-*N*e*O*mdU. The percentage of putT replacing T in phage phi W-14 was in the range of 47-48% (see Figure 4). We examined the ligation efficiency of restriction fragments from phage ViI and phi W-14 by T4 DNA ligase. *Nla*III- (CATG/) and *Fat*I- (/CATG) partially digested, or *Rsa*I (GT/AC) completely digested ViI restriction fragments were ligated at 16°C overnight. The sticky ends of *Nla*III and *Fat*I fragments were efficiently ligated, whereas the blunt-ended *Rsa*I fragments were ligated at a lower efficiency (Figure 5A). The *Sau*3AI- or *Mbo*I-digested (partial digestions) of phi W-14 restriction fragments were ligated efficiently indicated by the appearance of large concatenated DNA after ligation. Lower ligation efficiency was observed for blunt-ended *Rsa*I fragments (Figure 5B). We concluded that even though modified T could slow down restriction digestions by *Nla*III and *Fat*I for ViI genomic DNA, or by *Sau*3AI and *Mbo*I for phi W-14 DNA, the resulting restriction fragments can be efficiently ligated by T4 DNA ligase. The lower efficiency of *Rsa*I fragment ligation is most likely due to the blunt-ended nature of the ligation (Sambrook et al., 1989; Tsai et al., 2017).

### Exonuclease Digestion of ViI and phi W-14 Genomic DNA

We next examined exonuclease activity on phage M6, ViI and phi W-14 DNA. Two types of phage DNA restriction fragments were digested with different amount of λ exonuclease or *E. coli* exonuclease III. Phage M6 and ViI restriction fragments were equally degraded by the two exonucleases (Supplementary Figures 7A,B). However, phi W-14 restriction fragments showed apparent slowed-down in exonuclease degradation (at 10–20 U range vs. 0.5 μg DNA) (Supplementary Figure 7C). Unmodified 2-log DNA ladder is sensitive to *E. coli* exonuclease III and λ exonuclease digestions (data not shown). The mechanism of phi W-14 DNA partial resistance to exonuclease digestion is still unknown. It was reported previously that the rate of DNA hydrolysis by non-specific endonuclease of modified phage PBS1 (dT substituted by dU) was decreased by 14.3-fold, and hypermodified phage T4 DNA also shows slow-down in nuclease degradation (Huang et al., 1982).

### Digestion of Phage ViI and phi W-14 DNA With DNA Glycosylase and AP Endonuclease

5hmdU can be excised by DNA repair enzymes AlkA and Mug from *E. coli*, and by human SMUG1 (hSMUG1) and TDG to create AP sites (apurinic/apyrimidinic site), which can be further cleaved by AP endonucleases (Ulbert et al., 2004). Since ViI genomic DNA contains a small amount of 5hmdU we tested whether ViI and phi W-14 genomic DNA could be fragmented by hSMUG1 and AP endonuclease. Supplementary Figure 8 shows that a small amount of smearing of ViI gDNA after treatment with hSMUG1 and Endonuclease VIII, probably resulting from cleavage in the small percentage of 5hmdU in the genome. Phi W-14 and λ DNA (a negative control) is quite resistant to the cleavage by the combination of these two enzymes. In the positive control sample, phage SP8 DNA (5hmdU substituted for T) was extensively hydrolyzed by hSMUG1 and Endonuclease VIII.

## Discussion

### Biological Function of Base Modification (nt Substitution)

In bacterial host and phage coevolution, phage use extensive base modifications (nt substitutions) to protect its genome against host restrictions. The results presented here demonstrate that hypermodified T derived from 5hmdU can also efficiently protect phage genomes against Type II restrictions, in analogous manner to modified Gs, such as dG^+^ found in phage 9g genome (Thiaville et al., 2016) and 2′-deoxy-7-amido-7-deazaguanosine (dADG) found in certain bacteria genomic islands (Yuan et al., 2018), to modified As, such as *N*6-(1-acetamido)-adenine in phage Mu genome (Hattman, 1979), to modified Cs, such as 5gmC in phage T4, 5hmC in phage T4gt, and 5mC in phage XP12 genome. Although not much *in vivo* restriction study has been carried out on T-hypermodified phages, it is very likely that there is a strong correlation between *in vitro* and *in vivo* restriction activity. Depending on the *in vivo* enzyme activity and level of restriction gene expression, restriction of phage infection can be in the range of 10^2^–10^6^ fold (reviewed in Pingoud et al., 2016). In this work we focus on Type II restrictions *in vitro*. Resistance against Type I restriction has not been studied and we only tested one ATP-dependent Type III restriction (*Eco*P15I, CAGCAG N25/). We hypothesize that phages M6, ViI, and phi W-14 may be resistant or partially resistant to Type I restriction as long as the restriction sites of these enzymes contain one or more TS dinucleotide sequence. 5mC-dependent REases are not tested on the three phage DNA substrates.

To counter adenine or cytosine modifications of phage genomes, bacteria develop modification-dependent REases (MDRE) to specifically attack modified DNA (Fleischman et al., 1976; Raleigh et al., 1989). For example, the *E. coli* GmrSD endonuclease attacks 5hmC and 5gmC modified DNA (Bair and Black, 2007; He et al., 2015). We have not found MDREs against modified T or modified G, but such enzymes might exist in nature. In addition, phages use anti-restriction proteins, small inhibitor proteins, DNA mimic protein to inhibit host-encoded REase (Rifat et al., 2008). Another likely function of modified bases is to help phage DNA packaging; for example, the positive charges of protonated -NH_2_ groups in the putrescinyl group of putT side chain helps counter balance the negative charges of the DNA backbone, thereby enhancing DNA structural flexibility and denser packing the DNA into the viral capsid (Scraba et al., 1983). In addition to enhanced DNA packing capability, modified bases have also been implicated in regulation of promoter strength and gene expression during initiation of DNA packaging into phage prohead (Greene et al., 1986). This has been demonstrated in phage P1 that the GATC sequences in the packaging site (*pac*) are recognized and methylated by the phage-encoded Dam MTase triggering cleavage of *pac* sites and phage packaging initiation (Coulby and Sternberg, 1987; Sternberg and Coulby, 1990). The effect of modified Ts on promoter strength and transcription regulation remains to be studied for the three phages reported here.

*Bacillus* phage SPO1 genomic DNA wherein >98% of Ts are replaced by 5hmdU is resistant or partially resistant to over 50% of Type II REases with 0–4 Ts in the recognition sequences (Huang et al., 1982; Vilpo and Vilpo, 1995). Another important aspect of the non-canonical nucleotide in the genome is the regulation of viral gene expression: temporal differential expression of the early and late viral genes in transcription (Greene et al., 1986; Hoet et al., 1992). Phage M6 DNA carrying the modified base 5-aminoethyl which confers slightly higher resistance (48.9% complete and 15.8% partial resistance). More complex modifications, such as in phage ViI led to even higher resistance level (∼71.0%). It is not clear how phages balance the need for base modification to become highly resistant to host-encoded restrictions and energy (ATP) consumption on making these base modifications and the ultimate evolutionary advantage in successful infection of bacterial hosts. Some *Bacillus* phage or prophage genomes encode frequent multi-specificity cytosine MTases (Xu et al., 1992, 1997; Schumann et al., 1995). Phage T2 and T4 encode an adenine MTase (*dam*^+^) that methylates GATC sites to provide more resistance against REases with overlapping GATC sequence. T even phages provide examples of two types of base modifications (6mA+5hmC or 6mA+5gmC) in their genome (Schlagman and Hattman, 1983). We have not yet observed phage genomes having both modified cytosine and thymine perhaps because of the small sample size of the sequenced phage genomes. Phage λ DNA contains some modified cytosine (5mC) and adenine (6mA, ∼15%) when the phage is propagated on Dam^+^ Dcm^+^ *E. coli* host. With advancement in DNA sequencing technology, single molecule SMRT sequencing and Nanopore sequencing might be able to sequence and identify more modifies bases in addition to N4mC and N6mA in DNA (Flusberg et al., 2010; Clark et al., 2012).

### MDRE in *Pseudomonas* Strains and Phage Therapy

The *Pseudomonas* phage M6 DNA can be efficiently methylated by a few frequent C5-cytosine MTases to achieve double base modifications, which can provide more protection against Type II restrictions with GC recognition sequence. But the 5mC modifications also provide an opportunity for 5mC-dependent restrictions. Some *Pseudomonas* genomes encode *Mcr*BC and Mrr-like, and *Bis*I-like enzymes (REBASE) that remain to be characterized.

A cocktail of *Pseudomonas* lytic phages has been successfully used to treat *P. aeruginosa* infections in animal models (Forti et al., 2018). The DNA restriction data presented here suggests clinicians should take into consideration of heavily modified phage genomes and host restriction systems on the success or failure of phage-based therapies.

### Conserved Sequence Motif in Resistant Sites

Analysis of the resistant sites in the phage genomes revealed a conserved motif TG, TC, or TS, suggesting the modified Ts possess certain sequence specificity, which may have been conferred by phage DNA 5hmdU kinase that phosphorylates the base for further chemical modification. Understanding the enzymes involved in thymidine hypermodification in phage genomes is an active research topic in our lab (YJL, PW) (Lee et al., 2018). In support of the preferred TS specificity observed among the resistant sites, purified DNA 5hmdUMP kinase can phosphorylate the 5hmdU base in phage DNA substrates (*Nco*I, CCATGG) (PW, unpublished result). For complete restriction digestion of hypermodified T phage DNA and cloning of certain genes (restriction fragments), Supplementary Tables 1–3 provide a useful guidance to choose among various commercially available restriction enzymes.

### CRISPR-Cas Associated Protein Cas4 Nuclease and Homing Endonucleases

Both ViI and phi W-14 encode a three-gene cluster with predicted function in restriction (phage against phage superinfection). ORFs Vi01_137, 138, and 139 encode putative RNA-DNA and DNA-DNA helicase/ATPase, CRISPR-Cas associated protein Cas4 nuclease (Cas4 IA-ID, IIB superfamily), and ssDNA binding protein in the ViI genome. Similarly, a three-gene cluster in phi W-14 genome contains gp030, gp032, and gp031. But the exact function of the three proteins involved in DNA metabolism (restriction) is still unknown. Incidentally, phage ViI also encodes a superinfection exclusion protein (Vi01_111c) that may play a role in attenuation of other phage infections. ViI genome encodes one GIY-YIG superfamily endonuclease (Vi01_159c); and phi W-14 genome encodes two HNH endonucleases (gp143 and gp219). These endonucleases are probably homing endonucleases involved in insertion of intron into intronless target known as intron “homing” since there are no cognate MTase genes associated with the predicted endonucleases (reviewed in Stoddard and Belfort, 2010). Because of large recognition sequence of homing endonucleases (typically 16–30 bp), there is no need to encode cognate MTase for self-protection.

### Base J and 5hmdU in Eukaryotic Parasite, DNA Glycosylase/AP Endonuclease

Base J (*O*-linked glucosylated thymine, β-D-glucosyl-deoxymethyluracil) in human pathogens *Trypanosoma brucei*, *Trypanosoma cruzi*, and *Leishmania* species, consisted of about 1% of total T in the genomes. The modified base J is an important regulatory epigenetic mark in trypanosomatids to influence gene expression. The JBP1/2 enzymes catalyze hydroxylation of thymine (Yu et al., 2007), forming 5-hydroxymethyluracil (5hmdU), which is then glucosylated by the base J-associated glucosyltransferase (JGT). The presence of glucosylated 5hmdU has not been reported in phage genomes. DNA with base J modification is not a substrate for DNA repair enzyme AlkA and Mug of *E. coli*, and hSMUG1 and TDG (Ulbert et al., 2004). When phage genomes contain large number of 5hmdU bases, the phage DNA is possibly subjected to DNA glycosylase cleavage. We show that phage ViI gDNA can be partially digested by hSMUG1 and Endonuclease VIII due to the presence of small amount of 5hmdU in the genome. But phi W-14 is fully resistant to hSMUG1 and endonuclease VIII; while phage SP8 genome with 5hmdU is heavily degraded by the two enzymes. The 5hmdU base is to be further modified to become resistant to host DNA glycosylases/AP endonucleases and REases such as in the case of phage M6, ViI and phi W-14. Alternatively, the 5hmdU-containing phages can only infect bacterial hosts deficient in AlkA- and Mug-like repair enzymes or by expression of phage-encoded enzyme inhibitors.

## Data Availability

The datasets generated for this study are available on request to the corresponding author.

## Author Contributions

KF, RT, MX, IC, AC, and S-YX performed experimental work. M-QX, Y-JL, IC, PW, and S-YX contributed with ideas. S-YX wrote the manuscript with input from all the authors.

## Conflict of Interest Statement

Y-JL, M-QX, IC, PW, and S-YX are employees of New England Biolabs, Inc. New England Biolabs commercializes reagents for molecular biological applications. The remaining authors declare that the research was conducted in the absence of any commercial or financial relationships that could be construed as a potential conflict of interest.
