# Supplementary material for: Type II Restriction of Bacteriophage DNA With 5hmdU-Derived Base Modifications
Source: Front Microbiol. 2019 Mar 29;10:584. doi: 10.3389/fmicb.2019.00584 (PMC6449724; doi:10.3389/fmicb.2019.00584)
Supplement: Supplementary file 1 [file Table_1.docx]

**Supplementary Material**

**Supplementary Tables 1-3; Supplementary Figures 1-8**

**Type II Restriction of Bacteriophage DNA with 5hmdU-derived Base Modifications**

Kiersten Flodman#, Rebecca Tsai#, Michael Y. Xu#, Ivan R. Corrêa Jr., Alyssa Copelas, Yan-Jiun Lee, Ming-Qun Xu, Peter Weigele, and Shuang-yong Xu*

New England Biolabs, Inc. 240 County Road, Ipswich, MA 01938, USA

#Authors contributed equally to this work

*Corresponding author

Dr. Shuang-yong Xu

[xus@neb.com](mailto:xus@neb.com)

Telephone: 1-978-380-7287

Fax: 1-978-921-1350

Key words: Type II restriction and modification, 5hmdU-derived nucleotide modification, *Pseudomonas* bacteriophage (phage) M6, *Salmonella* phage ViI (Vi1), *Delftia* phi W-14.

**Supplementary Table 1. Type II restriction of phage M6 genomic DNA (gDNA).**

Type II and III restriction endonucleases (REases) were supplied by NEB. Type IIM and IV modification-dependent REases (MDRE) and strand-specific DNA nicking enzymes were not tested. c, complete digestion; p, partial digestion; vp, very partial digestion; x, resistance; no site, restriction site absent. Phage M6 gDNA show resistance against 48.4% of Type II restrictions tested here. R=A or G, Y=C or T, M=A or C, K=G or T, S=C or G, W=A or T, N=A, C, G, or T.

| **REase** | **Recognition Sequence** | **# of T** | **Cut Status** | **TN Dinucleotides** |
| --- | --- | --- | --- | --- |
| AatII | GACGTC | 2 | x | TC, TG |
| AccI | GTMKAC | 2 to 4 | no site |  |
| Acc65I | GGTACC | 2 | vp | TG, TA |
| AciI | CCGC | 0 | c |  |
| AclI | AACGTT | 4 | no site |  |
| AcuI | CTGAAGN16/N14 | 3 | x | TC, TG, TT |
| AfeI | AGCGCT | 2 | x | TN |
| Af1II | CTTAAG | 4 | vp | TC, TT, TA |
| Af1III | ACRYGT | 2 to 4 | x | TG |
| AgeI-HF | ACCGGT | 2 | no site |  |
| AhdI | GAC N5 GTC | 2 | p |  |
| AleI | CAC N4 GTG | 2 | x | TC, TG |
| AluI | AGCT | 2 | p |  |
| AlwI | GGATCN4/N5 | 2 | c |  |
| AlwNI | CAG N3 CTG | 2 | x | TC, TG |
| ApaI | GGGCCC | 0 | x | TG |
| ApaLI | GTGCAC | 2 | x | TG |
| ApeKI | GCWGC | 1 | c |  |
| ApoI | RAATTY | 4 to 6 | c |  |
| AscI | GGCGCGCC | 0 | no site |  |
| AseI | ATTAAT | 6 | no site |  |
| AsiSI | GCGATCGC | 2 | no site |  |
| AvaI | CYCGRG | 0 to 2 | c |  |
| AvaII | GGWCC | 0 to 1 | p |  |
| AvrII | CCTAGG | 2 | no site |  |
| BaeI | N10/N15AC N4 GTAYCN12/N7 | 3 to 4 | x | TC, TA |
| BaeGI | GKGCMC | 0 to 2 | x | TG |
| BamHI-HF | GGATCC | 2 | no site |  |
| BanI | GGYRCC | 0 to 2 | p |  |
| BanII | GRGCYC | 0 to 2 | c |  |
| BbsI | GAAGACN2/N6 | 3 | x | TC, TG |
| BbvI | GCAGCN8/N12 | 1 | x | TG |
| BbvCI | CCTCAGC | 2 | x | TC, TG |
| BccI | CCATCN4/N5 | 2 | x | TC, TG |
| BceAI | ACGGCN12/N14 | 1 | c |  |
| BcgI | N10/N12CGA N6 TGCN12/N10 | 2 | x | TC, TG |
| BciVI | GTATCCN6/N5 | 3 | p |  |
| Bc1I | TGATCA | 4 | x | TC, TG |
| BcoDI | GTCTCN1/N5 | 2 | p |  |
| BfaI | CTAG | 2 | c |  |
| BfuAI | ACCTGCN4/N8 | 2 | x | TG, TA |
| BfuCI | GATC | 2 | p |  |
| Bg1I | GCC N5 GGC | 0 | p |  |
| Bg1II | AGATCT | 4 | no site |  |
| BlpI | GCTNAGC | 2 | c |  |
| BmgBI | CACGTC | 2 | x | TC, TG |
| BmrI | ACTGGGN5/N4 | 2 | x | TG, TA |
| BmtI-HF | GCTAGC | 2 | no site |  |
| BpmI | CTGGAGN16/N14 | 2 | x | TC, TG |
| Bpu10I | CCTNAGC | 2 | p |  |
| BpuEI | CTTGAGN16/N14 | 3 | x | TC, TG, TT |
| BsaI-HF | GGTCTCN1/N5 | 2 | vp | TC, TG |
| BsaAI | YACGTR | 2 to 4 | x | TG, TA |
| BsaBI | GAT N4 ATC | 4 | x | TC, TG, TN |
| BsaHI | GRCGYC | 0 to 2 | p |  |
| BsaJI | CCNNGG | 0 | c |  |
| BsaWI | WCCGGW | 2 | c |  |
| BsaXI | N9/N12AC N5 CTCCN10/N7 | 2 | p |  |
| BseRI | GAGGAGN10/N8 | 2 | p |  |
| BseYI | CCCAGC | 1 | p |  |
| BsgI | GTGCAGN16/N14 | 2 | x | TG |
| BsiEI | CGRYCG | 0 to 2 | c |  |
| BsiHKAI | GWGCWC | 2 | x | TC, TG |
| BsiWI | CGTACG | 2 | no site |  |
| BslI | CC N7 GG | 0 | c |  |
| BsmI | GAATGCN/ | 3 | x | TC, TG, TT |
| BsmAI | GTCTCN1/N5 | 2 | c |  |
| BsmBI | CGTCTCN1/N5 | 2 | p |  |
| BsmFI | GGGACN10/N14 | 1 | c |  |
| BsoBI | CYCGRG | 0 to 2 | x | TC |
| Bsp12861 | GDGCHC | 0 to 2 | c |  |
| BspCNI | CTCAGN10/N8 | 2 | vp | TC, TG |
| BspDI | ATCGAT | 4 | no site |  |
| BspEI | TCCGGA | 2 | no site |  |
| BspHI | TCATGA | 4 | x | TC, TG |
| BspMI | ACCTGCN4/N8 | 2 | x | TG, TN |
| BspQI | GCTCTTCN1/N4 | 3 | x | TC, TG, TT |
| BsrI | ACTGGN/ | 2 | vp | TG, TA |
| BsrBI | CCGCTC | 1 | c |  |
| BsrDI | GCAATGNN/ | 3 | x | TG, TT |
| BsrFI | RCCGGY | 0 to 2 | c |  |
| BsrGI-HF | TGTACA | 4 | x | TG, TA |
| BssHII | GCGCGC | 0 | c |  |
| BssS_a_I | CACGAG | 2 | x | TC, TG |
| BstAPI | GCA N5 TGC | 2 | x | TG |
| BstBI | TTCGAA | 4 | no site |  |
| BstEII-HF | GGTNACC | 2 | x | TG, TN |
| BstNI | CCWGG | 1 | x | TC, TG |
| BstUI | CGCG | 0 | c |  |
| BstXI | CCA N6 TGG | 2 | x | TC, TG |
| BstYI | RGATCY | 2 to 4 | no site |  |
| BstZI7I | GTATAC | 4 | no site |  |
| Bsu36I | CCTNAGG | 2 | no site |  |
| BtgI | CCRYGG | 0 to 2 | p |  |
| BtgZI | GCGATGN10/N14 | 2 | vp | TC, TG |
| BtsaI | GCAGTGNN/ | 2 | x | TG |
| BtsIMUTI | CAGTGNN/ | 2 | x | TG |
| BtscI | GGATGNN/ | 2 | x | TC, TG |
| Cac8I | GCNNGC | 0 | c |  |
| ClaI | ATCGAT | 4 | no site |  |
| CspCI | N10/N12CAAN5GTGGN12/N10 | 3 | x | TG, TT |
| CviAII | CATG | 2 | x | TC, TG |
| CviKI-I | RGCY | 0 to 2 | c |  |
| CviQI | GTAC | 2 | p |  |
| DdeI | CTNAG | 2 | p |  |
| DpnII | GATC | 2 | c |  |
| DraI | TTTAAA | 6 | no site |  |
| DraIII-HF | CAC N3 GTG | 2 | x | TC, TG |
| DrdI | GAC N6 GTC | 2 | p |  |
| EaeI | YGGCCR | 0 to 2 | p |  |
| EagI | CGGCCG | 0 | no site |  |
| EarI | CTCTTCN1/N4 | 3 | x | TC, TT |
| EciI | GGCGGAN11/N9 | 1 | c |  |
| Eco53kI | GAGCTC | 2 | x | TC, TG |
| EcoNI | CCT N5 AGG | 2 | c |  |
| EcoO109I | RGGNCCY | 0 to 2 | c |  |
| EcoP15I | CAGCAGN25/N27 | 2 | x | TC, TG |
| EcoRI-HF | GAATTC | 4 | vp | TC, TG, TT |
| EcoRV-HF | GATATC | 4 | no site |  |
| FatI | CATG | 2 | x | TC, TG |
| FauI | CCCGCN4/N6 | 0 | c |  |
| Fnu4HI | GCNGC | 0 | c |  |
| FokI | GGATGN9/N13 | 2 | c |  |
| FseI | GGCCGGCC | 0 | no site |  |
| FspI | TGCGCA | 2 | x | TG |
| HaeII | RGCGCY | 0 to 2 | p |  |
| HaeIII | GGCC | 0 | c |  |
| HgaI | GACGCN5/N10 | 1 | c |  |
| HhaI | GCGC | 0 | c |  |
| HincII | GTYRAC | 2 to 4 | x | TC, TG, TT |
| HindIII-HF | AAGCTT | 4 | no site |  |
| HinfI | GANTC | 2 | c |  |
| HinP1I | GCGC | 0 | c |  |
| HpaI | GTTAAC | 4 | no site |  |
| HpaII | CCGG | 0 | c |  |
| HphI | GGTGAN8/N7 | 2 | x | TC, TG |
| Hpy99I | CGWCG | 0 to 1 | c |  |
| Hpy166II | GTNNAC | 2 | x | TG, TN |
| Hpy188I | TCNGA | 2 | c |  |
| Hpy188III | TCNNGA | 2 | p |  |
| HpyAV | CCTTCN6/N5 | 2 | c |  |
| HpyCH4III | ACNGT | 2 | p |  |
| HpyCH4IV | ACGT | 2 | c |  |
| HpyCH4V | TGCA | 2 | x | TG |
| KasI | GGCGCC | 0 | c |  |
| KpnI-HF | GGTACC | 2 | x | TG, TA |
| MboI | GATC | 2 | c |  |
| MboII | GAAGAN8/N7 | 3 | c |  |
| MfeI | CAATTG | 4 | x | TG, TT |
| MluI-HF | ACGCGT | 2 | no site |  |
| MluCI | AATT | 4 | c |  |
| MlyI | GAGTCN5/N5 | 2 | p |  |
| MmeI | TCCRACN20/N18 | 2 to 3 | p |  |
| MnlI | CCTCN7/N6 | 1 | c |  |
| MscI | TGGCCA | 2 | x | TG |
| MseI | TTAA | 4 | p |  |
| MslI | CAY N4 RTG | 2 to 4 | x | TG, TN |
| MspI | CCGG | 0 | c |  |
| MspA1I | CMGCKG | 0 to 2 | p |  |
| MwoI | GC N7 GC | 0 | c |  |
| NaeI | GCCGGC | 0 | c |  |
| NarI | GGCGCC | 0 | x | TG |
| NciI | CCSGG | 0 | c |  |
| NcoI-HF | CCATGG | 2 | x | TC, TG, |
| NdeI | CATATG | 4 | x | TC, TG, TA |
| NgoMIV | GCCGGC | 0 | vp | TG |
| NheI-HF | GCTAGC | 2 | no site |  |
| NlaIII | CATG | 2 | x | TC, TG |
| NlaIV | GGNNCC | 0 | c |  |
| NmeAIII | GCCGAGN20/N18 | 1 | Inconclusive |  |
| NotI-HF | GCGGCCGC | 0 | no site |  |
| NruI-HF | TCGCGA | 2 | no site |  |
| NsiI-HF | ATGCAT | 4 | no site |  |
| NspI | RCATGY | 2 to 4 | x | TG |
| PacI | TTAATTAA | 8 | no site |  |
| PaeR7I | CTCGAG | 2 | no site |  |
| PciI | ACATGT | 4 | x | TG |
| PflFI | GAC N3 GTC | 2 | vp | TC, TG |
| PflMI | CCA N5 TGG | 2 | x | TC, TG |
| PleI | GAGTCN4/N5 | 2 | p |  |
| PluTI | GGCGCC | 0 | x | TG |
| PmeI | GTTTAAAC | 6 | no site |  |
| PmlI | CACGTG | 2 | x | TC, TG |
| PpuMI | RGGWCCY | 1 to 3 | c |  |
| PshAI | GAC N3 GTC | 2 | vp | TC, TG |
| PsiI | TTATAA | 6 | no site |  |
| PspGI | CCWGG | 0 to 1 | vp | TC, TG |
| PspOMI | GGGCCC | 0 | x | TG |
| PspXI | VCTCGAGB | 2 to 4 | no site |  |
| PstI-HF | CTGCAG | 2 | x | TC, TG |
| PvuI-HF | CGATCG | 2 | no site |  |
| PvuII-HF | CAGCTG | 2 | x | TC, TG |
| RsaI | GTAC | 2 | c |  |
| RsrII | CGGWCCG | 1 | c |  |
| SacI-HF | GAGCTC | 2 | x | TC, TG |
| SacII | CCGCGG | 0 | c |  |
| SalI-HF | GTCGAC | 2 | no site |  |
| SapI | GCTCTTCN1/N4 | 3 | x | TC, TG, TT |
| Sau3AI | GATC | 2 | p |  |
| Sau96I | GGNCC | 0 | c |  |
| SbfI-HF | CCTGCAGG | 2 | no site |  |
| ScaI-HF | AGTACT | 4 | no site |  |
| ScrFI | CCNGG | 0 | c |  |
| SexAI | ACCWGGT | 3 | vp | TG, TN |
| SfaNI | GCATCN5/N9 | 2 | x | TC, TG |
| SfcI | CTRYAG | 2 to 4 | p |  |
| SfiI | GGCC N5 GGCC | 0 | Inconclusive |  |
| SfoI | GGCGCC | 0 | c |  |
| SgrAI | CRCCGGYG | 0 to 2 | c |  |
| SmaI | CCCGGG | 0 | c |  |
| SmlI | CTYRAG | 2 to 4 | vp | TC, TG |
| SnaBI | TACGTA | 4 | no site |  |
| SpeI-HF | ACTAGT | 4 | no site |  |
| SphI-HF | GCATGC | 2 | vp | TG |
| SrfI | GCCCGGGC | 0 | c |  |
| SspI-HF | AATATT | 6 | x | TA, TT |
| StuI | AGGCCT | 2 | c |  |
| StyI-HF | CCWWGG | 2 | x | TG, TT |
| StyD4I | CCNGG | 0 | c |  |
| SwaI | ATTTAAAT | 8 | no site |  |
| TaqI | TCGA | 2 | c |  |
| TfiI | GAWTC | 3 | p |  |
| TseI | GCWGC | 1 | x | TG |
| Tsp45I | GTSAC | 2 | x | TC, TG |
| TspMI | CCCGGG | 0 | c |  |
| TspRI | NNCASTGNN/ | 2 | p |  |
| Tth111I | GACNNNGTC | 2 | vp | TC |
| XbaI | TCTAGA | 4 | no site |  |
| XcmI | CCA N9 TGG | 2 | x | TG |
| XhoI | CTCGAG | 2 | no site |  |
| XmaI | CCCGGG | 0 | c |  |
| XmnI | GAA N4 TTC | 4 | vp | TC, TG, TT |
| ZraI | GACGTC | 2 | x | TC, TG |

**Supplementary Table 2**. Type II restriction of phage ViI (Vi1) genomic DNA. ViI gDNA is resistant to 71.0% of Type II restrictions examined here. R=A or G, Y=C or T, M=A or C, K=G or T, S=C or G, W=A or T, N=A, C, G, or T.

| **REase** | **Recognition Sequence** | **# of T** | **Cut Status** | **TN Dinucleotides** |
| --- | --- | --- | --- | --- |
| AatII | GACGTC | 2 | x | TC, TG |
| AccI | GTMKAC | 2 to 4 | p |  |
| Acc65I | GGTACC | 2 | x | TG, TA |
| AciI | CCGC | 0 | c |  |
| AclI | AACGTT | 4 | p |  |
| AcuI | CTGAAGN16/N14 | 3 | x | TC, TG, TT |
| AfeI | AGCGCT | 2 | p |  |
| Af1II | CTTAAG | 4 | x | TC, TT, TA |
| Af1III | ACRYGT | 2 to 4 | p |  |
| AgeI-HF | ACCGGT | 2 | p |  |
| AhdI | GAC N5 GTC | 2 | x | TC, TG |
| AleI | CAC N4 GTG | 2 | x | TC, TG |
| AluI | AGCT | 2 | p |  |
| AlwI | GGATCN4/N5 | 2 | x | TC, TG |
| AlwNI | CAG N3 CTG | 2 | x | TC, TG |
| ApaI | GGGCCC | 0 | no site |  |
| ApaLI | GTGCAC | 2 | x | TG |
| ApeKI | GCWGC | 1 | p |  |
| ApoI | RAATTY | 4 to 6 | vp | TG, TT |
| AscI | GGCGCGCC | 0 | no site |  |
| AseI | ATTAAT | 6 | vp | TT, TA |
| AvaI | CYCGRG | 0 to 2 | x | TC |
| AvaII | GGWCC | 0 to 1 | x | TC, TG |
| AvrII | CCTAGG | 2 | x | TC, TA |
| BaeI | N10/N15AC N4 GTAYCN12/N7 | 3 to 4 | x | TC, TA, TN |
| BaeGI | GKGCMC | 0 to 2 | x | TG |
| BamHI-HF | GGATCC | 2 | x | TC, TG |
| BanI | GGYRCC | 0 to 2 | x | TA, TG |
| BanII | GRGCYC | 0 to 2 | no site |  |
| BbsI | GAAGACN2/N6 | 3 | x | TC, TG, TT |
| BbvI | GCAGCN8/N12 | 1 | x | TG |
| BbvCI | CCTCAGC | 2 | x | TC, TG |
| BccI | CCATCN4/N5 | 2 | x | TC, TG |
| BceAI | ACGGCN12/N14 | 1 | p |  |
| BcgI | N10/N12CGA N6 TGCN12/N10 | 2 | x | TC, TG |
| BciVI | GTATCCN6/N5 | 3 | x | TC, TG, TA |
| Bc1I | TGATCA | 4 | x | TC, TG |
| BcoDI | GTCTCN1/N5 | 2 | x | TC, TG |
| BfaI | CTAG | 2 | p |  |
| BfuAI | ACCTGCN4/N8 | 2 | x | TG, TN |
| BfuCI | GATC | 2 | x | TC, TG |
| Bg1I | GCC N5 GGC | 0 | vp |  |
| Bg1II | AGATCT | 4 | x | TC, TN |
| BlpI | GCTNAGC | 2 | p |  |
| BmgBI | CACGTC | 2 | x | TC, TG |
| BmrI | ACTGGGN5/N4 | 2 | x | TG, TN |
| BmtI-HF | GCTAGC | 2 | x | TG, TA |
| BpmI | CTGGAGN16/N14 | 2 | x | TC, TG |
| Bpu10I | CCTNAGC | 2 | vp |  |
| BpuEI | CTTGAGN16 | 3 | x | TC, TG, TT |
| BsaI-HF | GGTCTCN1/N5 | 2 | x | TC, TG |
| BsaAI | YACGTR | 2 to 4 | p |  |
| BsaBI | GAT N4 ATC | 4 | x | TC, TG |
| BsaHI | GRCGYC | 0 to 2 | x | TC, TG |
| BsaJI | CCNNGG | 0 | p |  |
| BsaWI | WCCGGW | 2 | x | TC, TN |
| BsaXI | N9/N12AC N5 CTCCN10/N7 | 2 | x | TC, TN |
| BseRI | GAGGAGN10/N8 | 2 | x | TC |
| BseYI | CCCAGC | 1 | x | TC, TG |
| BsgI | GTGCAGN16/N14 | 2 | x | TG |
| BsiEI | CGRYCG | 0 to 2 | x | TC |
| BsiHKAI | GWGCWC | 2 | x | TC, TG |
| BsiWI | CGTACG | 2 | x | TC, TA |
| BslI | CC N7 GG | 0 | c |  |
| BsmI | GAATGCN/ | 3 | x | TC, TG, TT |
| BsmAI | GTCTCN1/N5 | 2 | x | TC, TG |
| BsmBI | CGTCTCN1/N5 | 2 | x | TC |
| BsmFI | GGGACN10/N14 | 1 | x | TC, TG |
| BsoBI | CYCGRG | 0 to 2 | x | TC |
| Bsp12861 | GDGCHC | 0 to 2 | p |  |
| BspCNI | CTCAGN10/N8 | 2 | x | TC, TG |
| BspDI | ATCGAT | 4 | x | TC, TA |
| BspEI | TCCGGA | 2 | x | TC |
| BspHI | TCATGA | 4 | x | TC, TG |
| BspMI | ACCTGCN4/N8 | 2 | x | TG, TN |
| BspQI | GCTCTTCN1/N4 | 3 | x | TC, TG, TT |
| BsrI | ACTGGN/ | 2 | vp | TG |
| BsrBI | CCGCTC | 1 | vp | TC |
| BsrDI | GCAATGNN/ | 3 | x | TG, TT |
| BsrFI | RCCGGY | 0 to 2 | x | TG |
| BsrGI-HF | TGTACA | 4 | x | TG, TA |
| BssHII | GCGCGC | 0 | vp | TG |
| BssS_a_I | CACGAG | 2 | x | TC, TG |
| BstAPI | GCA N5 TGC | 2 | x | TG |
| BstBI | TTCGAA | 4 | x | TC, TT |
| BstEII-HF | GGTNACC | 2 | x | TG, TN |
| BstNI | CCWGG | 1 | x | TC, TG |
| BstUI | CGCG | 0 | c |  |
| BstXI | CCA N6 TGG | 2 | x | TG |
| BstYI | RGATCY | 2 to 4 | x | TC |
| BstZI7I | GTATAC | 4 | vp | TG, TA |
| Bsu36I | CCTNAGG | 2 | vp | TC, TN |
| BtgI | CCRYGG | 0 to 2 | x | TG |
| BtgZI | GCGATGN10/N14 | 2 | x | TC, TG |
| BtsaI | GCAGTGNN/ | 2 | x | TG |
| BtsIMUTI | CAGTGNN/ | 2 | x | TC, TG |
| BtscI | GGATGNN/ | 2 | x | TC, TG |
| Cac8I | GCNNGC | 0 | p |  |
| ClaI | ATCGAT | 4 | x | TC, TN |
| CspCI | N10/N12CAAN5GTGGN12/N10 | 3 | x | TG, TT |
| CviAII | CATG | 2 | x | TC, TG |
| CviKI-I | RGCY | 0 to 2 | c |  |
| CviQI | GTAC | 2 | c |  |
| DdeI | CTNAG | 2 | p |  |
| DpnI | G(N6m)ATC | 2 | x | TC, TG |
| DpnII | GATC | 2 | x | TC, TG |
| DraI | TTTAAA | 6 | p |  |
| DraIII-HF | CAC N3 GTG | 2 | vp | TC, TG |
| DrdI | GAC N6 GTC | 2 | x | TC, TG |
| EaeI | YGGCCR | 0 to 2 | x | TG |
| EagI | CGGCCG | 0 | inconclusive | TC |
| EarI | CTCTTCN1/N4 | 3 | x | TC, TT |
| EciI | GGCGGAN11/N9 | 1 | p |  |
| Eco53kI | GAGCTC | 2 | no site |  |
| EcoNI | CCT N5 AGG | 2 | p |  |
| EcoO109I | RGGNCCY | 0 to 2 | x | TC |
| EcoP15I | CAGCAGN25/N27 | 2 | x | TC, TG |
| EcoRI-HF | GAATTC | 4 | x | TC, TG, TT |
| EcoRV-HF | GATATC | 4 | x | TC, TG, TA |
| FatI | CATG | 2 | p |  |
| FauI | CCCGCN4/N6 | 0 | c |  |
| Fnu4HI | GCNGC | 0 | c |  |
| FokI | GGATGN9/N13 | 2 | x | TC, TG |
| FseI | GGCCGGCC | 0 | no site |  |
| FspI | TGCGCA | 2 | x | TG |
| HaeII | RGCGCY | 0 to 2 | p |  |
| HaeIII | GGCC | 0 | c |  |
| HgaI | GACGCN5/N10 | 1 | x | TC, TG |
| HhaI | GCGC | 0 | c |  |
| HincII | GTYRAC | 2 to 4 | p |  |
| HindIII-HF | AAGCTT | 4 | x | TT, TN |
| HinfI | GANTC | 2 | x | TC, TG |
| HinP1I | GCGC | 0 | c |  |
| HpaI | GTTAAC | 4 | p |  |
| HpaII | CCGG | 0 | c |  |
| HphI | GGTGAN8/N7 | 2 | x | TC, TG |
| Hpy99I | CGWCG | 0 to 1 | p |  |
| Hpy166II | GTNNAC | 2 | c |  |
| Hpy188I | TCNGA | 2 | x | TC |
| Hpy188III | TCNNGA | 2 | x | TC |
| HpyAV | CCTTCN6/N5 | 2 | c |  |
| HpyCH4III | ACNGT | 2 | p |  |
| HpyCH4IV | ACGT | 2 | c |  |
| HpyCH4V | TGCA | 2 | x | TG |
| KasI | GGCGCC | 0 | inconclusive | TG |
| KpnI-HF | GGTACC | 2 | x | TG, TA |
| MboI | GATC | 2 | x | TC, TG |
| MboII | GAAGAN8/N7 | 3 | x | TC, TG, TT |
| MfeI | CAATTG | 4 | c |  |
| MluI-HF | ACGCGT | 2 | vp | TN |
| MluCI | AATT | 4 | c |  |
| MlyI | GAGTCN5/N5 | 2 | x | TC, TG |
| MmeI | TCCRACN20/N18 | 2 to 3 | x | TC, TT |
| MnlI | CCTCN7/N6 | 1 | x | TC |
| MscI | TGGCCA | 2 | x | TG |
| MseI | TTAA | 4 | c |  |
| MslI | CAY N4 RTG | 2 to 4 | vp | TC, TG |
| MspI | CCGG | 0 | p |  |
| MwoI | GC N7 GC | 0 | p |  |
| NaeI | GCCGGC | 0 | no site |  |
| NarI | GGCGCC | 0 | inconclusive | TG |
| NciI | CCSGG | 0 | inconclusive | TC |
| NcoI-HF | CCATGG | 2 | x | TA |
| NdeI | CATATG | 4 | x | TC, TG, TA |
| NgoMIV | GCCGGC | 0 | no site |  |
| NheI-HF | GCTAGC | 2 | p |  |
| NlaIII | CATG | 2 | p |  |
| NlaIV | GGNNCC | 0 | p |  |
| NmeAIII | GCCGAGN20/N18 | 1 | x | TC, TG |
| NotI-HF | GCGGCCGC | 0 | no site |  |
| NruI-HF | TCGCGA | 2 | x | TC |
| NsiI-HF | ATGCAT | 4 | x | TG, TA |
| NspI | RCATGY | 2 to 4 | x | TG, TN |
| PacI | TTAATTAA | 8 | x | TT, TA |
| PaeR7I | CTCGAG | 2 | x | TC |
| PciI | ACATGT | 4 | x | TG, TN |
| PflFI | GAC N3 GTC | 2 | x | TC, TG |
| PflMI | CCA N5 TGG | 2 | x | TG |
| PleI | GAGTCN4/N5 | 2 | x | TC |
| PluTI | GGCGCC | 0 | inconclusive | TG |
| PmeI | GTTTAAAC | 6 | vp | TG, TA, TT |
| PmlI | CACGTG | 2 | x | TC, TG |
| PpuMI | RGGWCCY | 1 to 3 | x | TC, TN |
| PshAI | GAC N3 GTC | 2 | x | TC, TG |
| PsiI | TTATAA | 6 | p |  |
| PspGI | CCWGG | 1 | x | TG |
| PspOMI | GGGCCC | 0 | no site |  |
| PspXI | VCTCGAGB | 2 to 4 | x | TC, TN |
| PstI-HF | CTGCAG | 2 | x | TC, TG |
| PvuI-HF | CGATCG | 2 | x | TC |
| PvuII-HF | CAGCTG | 2 | x | TC, TG |
| RsaI | GTAC | 2 | p |  |
| RsrII | CGGWCCG | 1 | x | TC |
| SacI-HF | GAGCTC | 2 | no site |  |
| SacII | CCGCGG | 0 | inconclusive | TC |
| SalI-HF | GTCGAC | 2 | x | TC, TG |
| SapI | GCTCTTCN1/N4 | 3 | x | TC, TG, TT |
| Sau3AI | GATC | 2 | x | TC, TG |
| Sau96I | GGNCC | 0 | p |  |
| SbfI-HF | CCTGCAGG | 2 | x | TC, TG |
| ScaI-HF | AGTACT | 4 | x | TA, TN |
| ScrFI | CCNGG | 0 | p |  |
| SexAI | ACCWGGT | 3 | x | TG, TN |
| SfaNI | GCATCN5/N9 | 2 | x | TC, TG |
| SfcI | CTRYAG | 2 to 4 | p |  |
| SfiI | GGCC N5 GGCC | 0 | no site |  |
| SfoI | GGCGCC | 0 | vp | TG |
| SgrAI | CRCCGGYG | 0 to 2 | x | TG |
| SmaI | CCCGGG | 0 | no site |  |
| SmlI | CTYRAG | 2 to 4 | x | TC, TG, TT |
| SnaBI | TACGTA | 4 | x | TA |
| SpeI-HF | ACTAGT | 4 | x | TA, TN |
| SphI-HF | GCATGC | 2 | x | TG |
| SrfI | GCCCGGGC | 0 | no site |  |
| SspI-HF | AATATT | 6 | p |  |
| StuI | AGGCCT | 2 | x | TN |
| StyI-HF | CCWWGG | 2 | x | TC, TG, TT |
| StyD4I | CCNGG | 0 | p |  |
| SwaI | ATTTAAAT | 8 | vp | TA, TT |
| TaqI | TCGA | 2 | c |  |
| TfiI | GAWTC | 3 | x | TC, TG, TT |
| TseI | GCWGC | 1 | p |  |
| Tsp45I | GTSAC | 2 | p |  |
| TspMI | CCCGGG | 0 | no site |  |
| TspRI | NNCASTGNN/ | 2 | p |  |
| Tth111I | GACNNNGTC | 2 | x | TC, TG |
| XbaI | TCTAGA | 4 | x | TC, TA, TT |
| XcmI | CCA N9 TGG | 2 | x | TC, TG |
| XhoI | CTCGAG | 2 | x | TC |
| XmaI | CCCGGG | 0 | no site |  |
| XmnI | GAA N4 TTC | 4 | x | TC, TG, TT |
| ZraI | GACGTC | 2 | x | TC, TG |

**Supplementary Table 3.** Type II restriction of phi W-14 genomic DNA. Phi W-14 gDNA is resistant to 68.8% of Type II restrictions tested here. R=A or G, Y=C or T, M=A or C, K=G or T, S=C or G, W=A or T, N=A, C, G, or T.

| **REase** | **Recognition Sequence** | **# of T's** | **Cut Status** | **TN Dinucleotides** |
| --- | --- | --- | --- | --- |
| AatII | GACGTC | 2 | x | TC, TG |
| AccI | GTMKAC | 2 to 4 | vp | TC, TG, TA |
| Acc65I | GGTACC | 2 | x | TG, TA |
| AciI | CCGC | 0 | c |  |
| AclI | AACGTT | 4 | vp | TA, TT |
| AcuI | CTGAAGN16/N14 | 3 | x | TC, TG, TT |
| AfeI | AGCGCT | 2 | vp | TN |
| Af1II | CTTAAG | 4 | no site |  |
| Af1III | ACRYGT | 2 to 4 | x | TG, TN |
| AgeI-HF | ACCGGT | 2 | x | TN |
| AhdI | GAC N5 GTC | 2 | x | TC, TG |
| AleI | CAC N4 GTG | 2 | x | TC, TG |
| AluI | AGCT | 2 | p |  |
| AlwI | GGATCN4/N5 | 2 | x | TC, TG |
| AlwNI | CAG N3 CTG | 2 | x | TC, TG |
| ApaI | GGGCCC | 0 | x | TG |
| ApaLI | GTGCAC | 2 | x | TG |
| ApeKI | GCWGC | 1 | c |  |
| ApoI | RAATTY | 4 to 6 | p |  |
| AseI | ATTAAT | 6 | x | TA, TT, TN |
| AvaI | CYCGRG | 0 to 2 | p |  |
| AvaII | GGWCC | 0 to 1 | c |  |
| AvrII | CCTAGG | 2 | x | TC, TA |
| BaeI | N10/N15AC N4 GTAYCN12/N7 | 3 to 4 | x | TC, TA, TN |
| BaeGI | GKGCMC | 0 to 2 | x | TG |
| BamHI-HF | GGATCC | 2 | x | TC, TG |
| BanI | GGYRCC | 0 to 2 | p |  |
| BanII | GRGCYC | 0 to 2 | p |  |
| BbsI | GAAGACN2/N6 | 3 | x | TC, TG, TT |
| BbvI | GCAGCN8/N12 | 1 | x | TG |
| BbvCI | CCTCAGC | 2 | x | TC, TG |
| BccI | CCATCN4/N5 | 2 | x | TC, TG |
| BceAI | ACGGCN12/N14 | 1 | p |  |
| BcgI | N10/N12CGA N6 TGCN12/N10 | 2 | x | TC, TG |
| BciVI | GTATCCN6/N5 | 3 | x | TC, TG, TA |
| Bc1I | TGATCA | 4 | x | TC, TG |
| BcoDI | GTCTCN1/N5 | 2 | p |  |
| BfaI | CTAG | 2 | c |  |
| BfuAI | ACCTGCN4/N8 | 2 | x | TG, TN |
| BfuCI | GATC | 2 | p |  |
| Bg1I | GCC N5 GGC | 0 | c |  |
| Bg1II | AGATCT | 4 | x | TC, TN |
| BlpI | GCTNAGC | 2 | x | TG, TN |
| BmgBI | CACGTC | 2 | x | TC, TG |
| BmrI | ACTGGGN5/N4 | 2 | x | TG, TN |
| BmtI-HF | GCTAGC | 2 | x | TG, TA |
| BpmI | CTGGAGN16/N14 | 2 | x | TC, TG |
| Bpu10I | CCTNAGC | 2 | x | TC, TN |
| BpuEI | CTTGAGN16 | 3 | x | TC, TG, TT |
| BsaI-HF | GGTCTCN1/N5 | 2 | x | TC, TG |
| BsaAI | YACGTR | 2 to 4 | x | TG, TA |
| BsaBI | GAT N4 ATC | 4 | x | TC, TG, TN |
| BsaHI | GRCGYC | 0 to 2 | p |  |
| BsaJI | CCNNGG | 0 | p |  |
| BsaWI | WCCGGW | 2 | vp | TC |
| BsaXI | N9/N12AC N5 CTCCN10/N7 | 2 | x | TC, TN |
| BseRI | GAGGAGN10/N8 | 2 | x | TC, TG |
| BseYI | CCCAGC | 1 | x | TC, TG |
| BsgI | GTGCAGN16/N14 | 2 | x | TG |
| BsiEI | CGRYCG | 0 to 2 | p |  |
| BsiHKAI | GWGCWC | 2 | x | TC, TG |
| BsiWI | CGTACG | 2 | x | TC, TA |
| BslI | CC N7 GG | 0 | p |  |
| BsmI | GAATGCN/ | 3 | x | TC, TG, TT |
| BsmAI | GTCTCN1/N5 | 2 | p |  |
| BsmBI | CGTCTCN1/N5 | 2 | vp | TC |
| BsmFI | GGGACN10/N14 | 1 | x | TC, TG |
| BsoBI | CYCGRG | 0 to 2 | p |  |
| Bsp12861 | GDGCHC | 0 to 2 | p |  |
| BspCNI | CTCAGN10/N8 | 2 | x | TC, TG |
| BspDI | ATCGAT | 4 | x | TC, TN |
| BspEI | TCCGGA | 2 | x | TC |
| BspHI | TCATGA | 4 | x | TC, TG |
| BspMI | ACCTGCN4/N8 | 2 | x | TG, TN |
| BspQI | GCTCTTCN1/N4 | 3 | x | TC, TG, TT |
| BsrI | ACTGGN/ | 2 | x | TG, TN |
| BsrBI | CCGCTC | 1 | vp | TC |
| BsrDI | GCAATGNN/ | 3 | x | TG, TT |
| BsrFI | RCCGGY | 0 to 2 | c |  |
| BsrGI-HF | TGTACA | 4 | x | TG, TA |
| BssHII | GCGCGC | 0 | p |  |
| BssS_a_I | CACGAG | 2 | x | TC, TG |
| BstAPI | GCA N5 TGC | 2 | x | TG |
| BstBI | TTCGAA | 4 | vp |  |
| BstEII-HF | GGTNACC | 2 | x | TG, TN |
| BstNI | CCWGG | 1 | x | TG |
| BstUI | CGCG | 0 | c |  |
| BstXI | CCA N6 TGG | 2 | x | TC, TG |
| BstYI | RGATCY | 2 to 4 | x | TC, TN |
| BstZI7I | GTATAC | 4 | x | TG, TA |
| Bsu36I | CCTNAGG | 2 | x | TC, TN |
| BtgI | CCRYGG | 0 to 2 | x | TC, TG |
| BtgZI | GCGATGN10/N14 | 2 | x | TC, TG |
| BtsaI | GCAGTGNN/ | 2 | x | TG |
| BtsIMUTI | CAGTGNN/ | 2 | x | TC, TG |
| BtscI | GGATGNN/ | 2 | vp | TC, TG |
| Cac8I | GCNNGC | 0 | p |  |
| ClaI | ATCGAT | 4 | x | TC, TN |
| CspCI | N10/N12CAAN5GTGGN12/N10 | 3 | x | TG, TT |
| CviAII | CATG | 2 | x | TG |
| CviKI-I | RGCY | 0 to 2 | c |  |
| CviQI | GTAC | 2 | p |  |
| DdeI | CTNAG | 2 | p |  |
| DpnI | G(N6m)ATC | 2 | x | TC, TG |
| DpnII | GATC | 2 | p |  |
| DraI | TTTAAA | 6 | x | TA, TT |
| DraIII-HF | CAC N3 GTG | 2 | x | TC, TG |
| DrdI | GAC N6 GTC | 2 | x | TC, TG |
| EaeI | YGGCCR | 0 to 2 | x | TG |
| EagI | CGGCCG | 0 | c |  |
| EarI | CTCTTCN1/N4 | 3 | x | TC, TT |
| EciI | GGCGGAN11/N9 | 1 | p |  |
| Eco53kI | GAGCTC | 2 | x | TC, TG |
| EcoNI | CCT N5 AGG | 2 | x | TC, TN |
| EcoO109I | RGGNCCY | 0 to 2 | p |  |
| EcoP15I | CAGCAGN25/N27 | 2 | x | TC, TG |
| EcoRI-HF | GAATTC | 4 | x | TC, TG, TT |
| EcoRV-HF | GATATC | 4 | x | TC, TG, TA |
| FatI | CATG | 2 | x | TC, TG |
| FauI | CCCGCN4/N6 | 0 | p |  |
| Fnu4HI | GCNGC | 0 | c |  |
| FokI | GGATGN9/13 | 2 | vp | TC, TG |
| FseI | GGCCGGCC | 0 | no site |  |
| FspI | TGCGCA | 2 | x | TG |
| HaeII | RGCGCY | 0 to 2 | p |  |
| HaeIII | GGCC | 0 | c |  |
| HgaI | GACGCN5/N10 | 1 | p |  |
| HhaI | GCGC | 0 | c |  |
| HincII | GTYRAC | 2 to 4 | vp | TG, TT |
| HindIII-HF | AAGCTT | 4 | no site |  |
| HinfI | GANTC | 2 | p |  |
| HinP1I | GCGC | 0 | c |  |
| HpaI | GTTAAC | 4 | vp | TG, TA, TT |
| HpaII | CCGG | 0 | c |  |
| HphI | GGTGAN8/N7 | 2 | x | TC, TG |
| Hpy99I | CGWCG | 0 to 1 | p |  |
| Hpy166II | GTNNAC | 2 | p |  |
| Hpy188I | TCNGA | 2 | x | TC |
| Hpy188III | TCNNGA | 2 | x | TC |
| HpyAV | CCTTCN6/N5 | 2 | p |  |
| HpyCH4III | ACNGT | 2 | vp | TN |
| HpyCH4IV | ACGT | 2 | p |  |
| HpyCH4V | TGCA | 2 | x | TG |
| KasI | GGCGCC | 0 | c |  |
| KpnI-HF | GGTACC | 2 | x | TG, TA |
| MboI | GATC | 2 | p |  |
| MboII | GAAGAN8/N7 | 3 | vp | TC, TG, TT |
| MfeI | CAATTG | 4 | x | TC, TG, TT |
| MluI-HF | ACGCGT | 2 | x | TN |
| MluCI | AATT | 4 | p |  |
| MlyI | GAGTCN5/N5 | 2 | p |  |
| MmeI | TCCRACN20/N18 | 2 to 3 | x | TC, TT |
| MnlI | CCTCN7/N6 | 1 | p |  |
| MscI | TGGCCA | 2 | x | TG |
| MseI | TTAA | 4 | p |  |
| MslI | CAY N4 RTG | 2 to 4 | x | TG, TN |
| MspI | CCGG | 0 | c |  |
| MwoI | GC N7 GC | 0 | c |  |
| NaeI | GCCGGC | 0 | x | TG |
| NarI | GGCGCC | 0 | p |  |
| NciI | CCSGG | 0 | c |  |
| NcoI-HF | CCATGG | 2 | x | TC, TG |
| NdeI | CATATG | 4 | x | TC, TG, TA |
| NgoMIV | GCCGGC | 0 | x | TG |
| NheI-HF | GCTAGC | 2 | x | TG, TA |
| NlaIII | CATG | 2 | x | TG |
| NlaIV | GGNNCC | 0 | p |  |
| NmeAIII | GCCGAGN20/N18 | 1 | x | TC, TG |
| NotI-HF | GCGGCCGC | 0 | x | TG |
| NruI-HF | TCGCGA | 2 | x | TC |
| NsiI-HF | ATGCAT | 4 | x | TG, TN |
| NspI | RCATGY | 2 to 4 | x | TG, TN |
| PacI | TTAATTAA | 8 | no site |  |
| PaeR7I | CTCGAG | 2 | x | TC |
| PciI | ACATGT | 4 | x | TG, TN |
| PflFI | GAC N3 GTC | 2 | x | TC, TG |
| PflMI | CCA N5 TGG | 2 | x | TC, TG |
| PleI | GAGTCN4/N5 | 2 | vp | TC, TG |
| PluTI | GGCGCC | 0 | p |  |
| PmeI | GTTTAAAC | 6 | x | TG, TA, TT |
| PmlI | CACGTG | 2 | x | TC, TG |
| PpuMI | RGGWCCY | 1 to 3 | x | TC, TN |
| PshAI | GAC N3 GTC | 2 | x | TC, TG |
| PsiI | TTATAA | 6 | vp | TA, TT |
| PspGI | CCWGG | 0 to 1 | vp | TC, TG |
| PspOMI | GGGCCC | 0 | x | TG |
| PspXI | VCTCGAGB | 2 to 4 | x | TC, TN |
| PstI-HF | CTGCAG | 2 | x | TC, TG |
| PvuI-HF | CGATCG | 2 | x | TC |
| PvuII-HF | CAGCTG | 2 | x | TC, TG |
| RsaI | GTAC | 2 | p |  |
| RsrII | CGGWCCG | 1 | x | TC |
| SacI-HF | GAGCTC | 2 | x | TC, TG |
| SacII | CCGCGG | 0 | Inconclusive | TC |
| SalI-HF | GTCGAC | 2 | x | TC |
| SapI | GCTCTTCN1/N4 | 3 | x | TC, TG, TT |
| Sau3AI | GATC | 2 | p |  |
| Sau96I | GGNCC | 0 | c |  |
| SbfI-HF | CCTGCAGG | 2 | x | TC, TG |
| ScaI-HF | AGTACT | 4 | x | TA, TN |
| ScrFI | CCNGG | 0 | c |  |
| SexAI | ACCWGGT | 3 | x | TG, TN |
| SfaNI | GCATCN5/N9 | 2 | x | TC, TG |
| SfcI | CTRYAG | 2 to 4 | x | TC, TG, TA |
| SfiI | GGCC N5 GGCC | 0 | Inconclusive | TG |
| SfoI | GGCGCC | 0 | c |  |
| SgrAI | CRCCGGYG | 0 to 2 | vp | TC, TG |
| SmaI | CCCGGG | 0 | p |  |
| SmlI | CTYRAG | 2 to 4 | x | TC, TG, TT |
| SnaBI | TACGTA | 4 | x | TA, TT |
| SpeI-HF | ACTAGT | 4 | x | TA, TN |
| SphI-HF | GCATGC | 2 | x | TG |
| SspI-HF | AATATT | 6 | x | TA, TT, TN |
| StuI | AGGCCT | 2 | x | TN |
| StyI-HF | CCWWGG | 2 | x | TC, TG, TA, TT |
| StyD4I | CCNGG | 0 | c |  |
| SwaI | ATTTAAAT | 8 | no site |  |
| TaqI | TCGA | 2 | p |  |
| TfiI | GAWTC | 3 | vp | TC, TG, TT |
| TseI | GCWGC | 1 | c |  |
| Tsp45I | GTSAC | 2 | x | TC, TG |
| TspMI | CCCGGG | 0 | p |  |
| TspRI | NNCASTGNN/ | 2 | c |  |
| Tth111I | GACNNNGTC | 2 | x | TC, TG |
| XbaI | TCTAGA | 4 | x | TC, TA |
| XcmI | CCA N9 TGG | 2 | x | TC, TG |
| XhoI | CTCGAG | 2 | x | TC |
| XmaI | CCCGGG | 0 | p |  |
| XmnI | GAA N4 TTC | 4 | vp | TC, TG, TT |
| ZraI | GACGTC | 2 | x | TC, TG |

**Supplementary Figure 1.**

**Chemical structures of phage nucleotide modifications (nt substitutions) discussed in this work.**

*Salmonella* phage ViI (Vi1) with 5-(2-aminoethoxy)methyldU (5-*N*e*O*mdU), *Pseudomonas* phage M6 DNA with 5-(2-aminoethyl)dU (5-*N*edU) [2], *Delftia* phage phi W-14 with α-putrescinylthymine (putT) [3], and *Bacillus* phage SP8 with 5-hydroxymethyldU (5hmdU) [4].

**

**

**Supplementary Figure 2.**

**Phage gDNA resistance/sensitivity to Type II restrictions presented by “pie” charts from the same restriction data shown in Supplementary Tables 1, 2, and 3.**

**Supplementary Figure 3.**

**Phage M6 restriction patterns generated by NEBcutter (assuming no base modification) [5].**

**
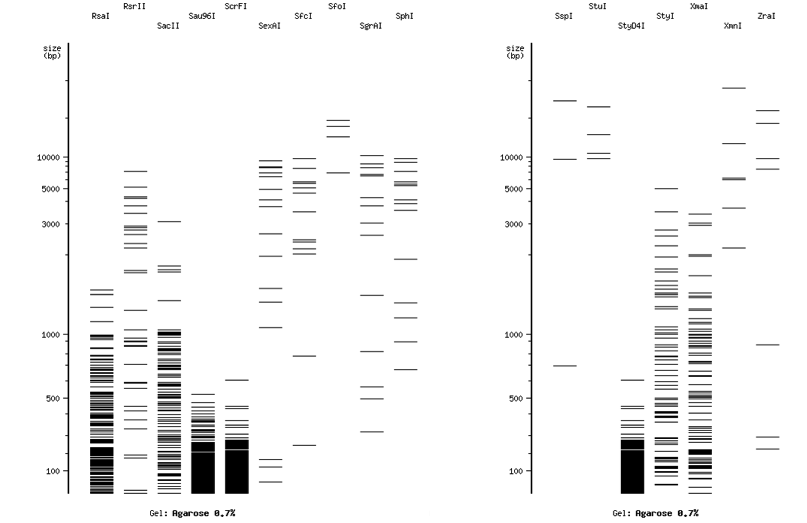
**

**Supplementary Figure 4.**

**Phage ViI (Vi1) restriction patterns generated by NEBcutter (assuming no base modification).**

**
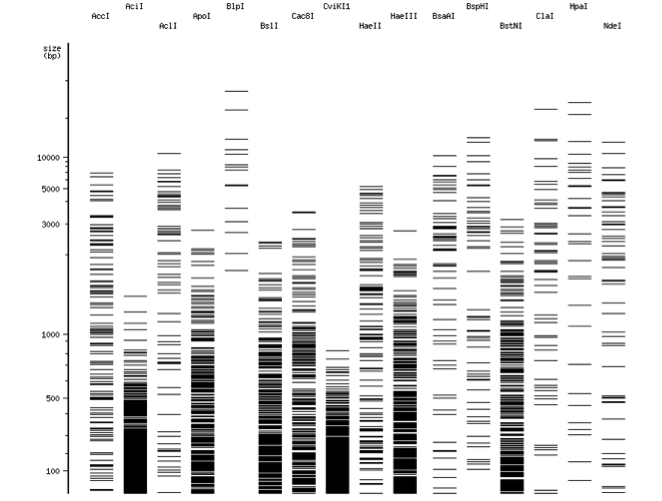
**

**Supplementary Figure 5.**

**Phi W-14 restriction patterns generated by NEBcutter (assuming no base modification).**

**
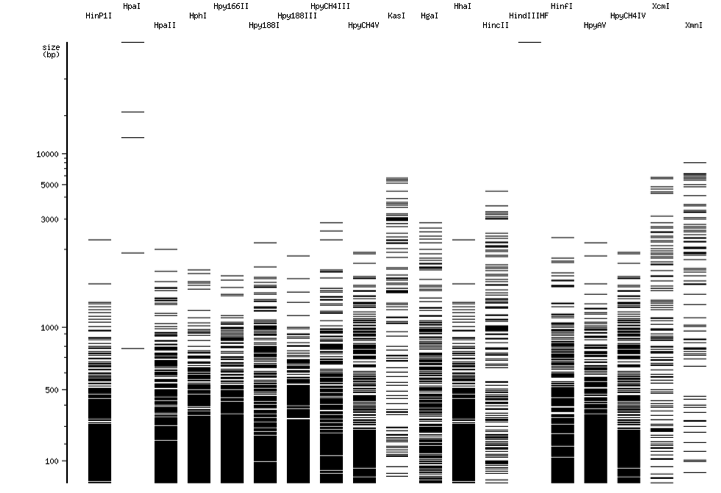
**

**Supplementary Figure 6.**

**Restriction of phage M6 and phi W-14 DNA with REases recognizing 6-bp sites with only GC sequence.**

**6A.** **Restriction of phage M6 gDNA with ApaI, PluTI, NarI, or PspOMI**. λ and pTYB2 DNA (unmodified) were digested as controls. The NEBcutter-generated restriction patterns in the absence of modification are shown to the right. pTYB2 contains one site each for ApaI, PluTI, NarI, and PspOMI. The sequence and map of pTYB2 can be found at www.neb.com.


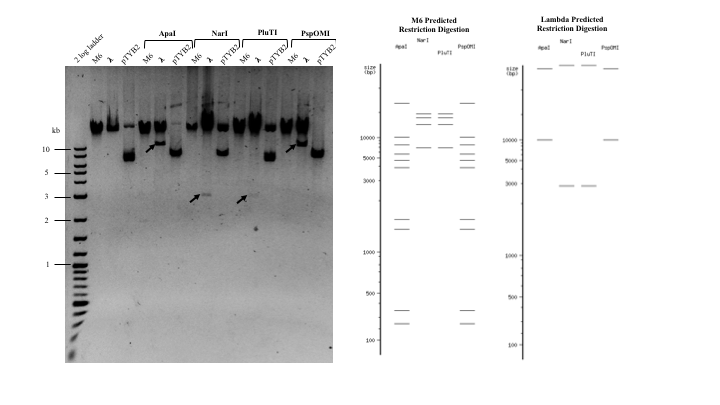


**6B.** **Restriction of phi W-14 gDNA with ApaI, NaeI, NgoMIV, NotI, or PspOMI respectively.** Phage λ and pTYB2 DNA (unmodified) were used as controls. Plasmid pTYB2 contains one NotI site, but λ DNA does not. Phi W-14 gDNA is mostly resistant to ApaI, NaeI, NgoMIV, NotI, or PspOMI restriction. NaeI and NgoMIV were able to cleave plasmid pTYB2, but unable to cut phi W-14 DNA.

**
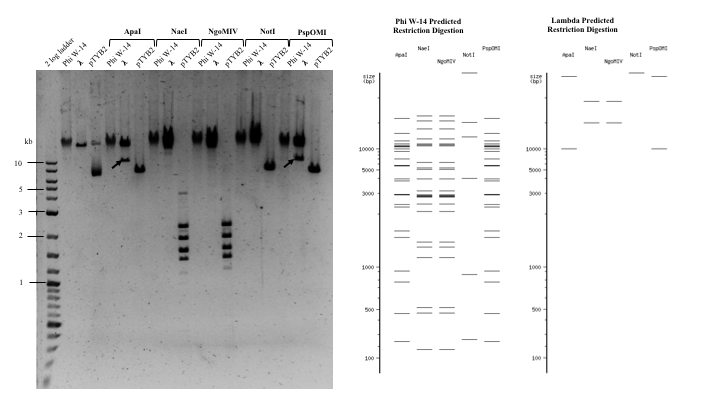
**

**Supplementary Figure 7. Phage λ exonuclease or *E. coli* exonuclease III digestion of phage DNAs. A)** phage M6 gDNA Cas8I and KasI restriction fragments digested by *E. coli* exonuclease III or λ exonuclease. **B)** phage ViI (Vi1) gDNA FauI and MfeI restriction fragments digested by *E. coli* exonuclease III or λ exonuclease. **C)** Phi W-14 gDNA BglI and EagI restriction fragments digested by *E. coli* exonuclease III or λ exonuclease.

**Supplementary Figure 8. Digestion of phage DNA with hSMUG1 and *E. coli* endonuclease VIII.** Phage DNA was first incubated with hSMUG1 for 1 h at 37^o^C, and then digested with Endonuclease VIII (10 U). Phage SP8 gDNA contains 5hmdU replacing all dT [4].

**Reference**

[1] K. Wilhelm, and W. Ruger, Deoxyuridylate-hydroxymethylase of bacteriophage SPO1. Virology 189 (1992) 640-6.

[2] Y.J. Lee, N. Dai, S.E. Walsh, S. Muller, M.E. Fraser, K.M. Kauffman, C. Guan, I.R. Correa, Jr., and P.R. Weigele, Identification and biosynthesis of thymidine hypermodifications in the genomic DNA of widespread bacterial viruses. Proc Natl Acad Sci U S A 115 (2018) E3116-E3125.

[3] A.M. Kropinski, R.J. Bose, and R.A. Warren, 5-(4-Aminobutylaminomethyl)uracil, an unusual pyrimidine from the deoxyribonucleic acid of bacteriophage phiW-14. Biochemistry 12 (1973) 151-7.

[4] P.P. Hoet, M.M. Coene, and C.G. Cocito, Replication cycle of *Bacillus subtilis* hydroxymethyluracil-containing phages. Annu Rev Microbiol 46 (1992) 95-116.

[5] T. Vincze, J. Posfai, and R.J. Roberts, NEBcutter: A program to cleave DNA with restriction enzymes. Nucleic Acids Res 31 (2003) 3688-91.
